# Direct and indirect Z-scheme heterostructure-coupled photosystem enabling cooperation of CO_2_ reduction and H_2_O oxidation

**DOI:** 10.1038/s41467-020-16742-3

**Published:** 2020-06-16

**Authors:** Ying Wang, Xiaotong Shang, Jinni Shen, Zizhong Zhang, Debao Wang, Jinjin Lin, Jeffrey C. S. Wu, Xianzhi Fu, Xuxu Wang, Can Li

**Affiliations:** 1 State Key Laboratory of Photocatalysis on Energy and Environment, Research Institute of Photocatalysis, College of Chemistry, Fuzhou University, 350108 Fuzhou, China; 2 Key Lab of Inorganic Synthetic and Applied Chemistry, State Key Lab Base of Eco-Chemical Engineering, College of Chemistry and Molecular Engineering, Qingdao University of Science & Technology, 266042 Qingdao, China; 3 Department of Chemical Engineering, National Taiwan University, 10617 Taipei, Taiwan; 4 State Key Laboratory of Catalysis, Dalian Institute of Chemical Physics, Chinese Academy of Sciences, 116023 Dalian, China

**Keywords:** Photocatalysis, Artificial photosynthesis, Solar fuels, Photocatalysis

## Abstract

The stoichiometric photocatalytic reaction of CO_2_ with H_2_O is one of the great challenges in photocatalysis. Here, we construct a Cu_2_O-Pt/SiC/IrO_x_ composite by a controlled photodeposition and then an artificial photosynthetic system with Nafion membrane as diaphragm separating reduction and oxidation half-reactions. The artificial system exhibits excellent photocatalytic performance for CO_2_ reduction to HCOOH and H_2_O oxidation to O_2_ under visible light irradiation. The yields of HCOOH and O_2_ meet almost stoichiometric ratio and are as high as 896.7 and 440.7 μmol g^−1^ h^−1^, respectively. The high efficiencies of CO_2_ reduction and H_2_O oxidation in the artificial system are attributed to both the direct Z-scheme electronic structure of Cu_2_O-Pt/SiC/IrO_x_ and the indirect Z-scheme spatially separated reduction and oxidation units, which greatly prolong lifetime of photogenerated electrons and holes and prevent the backward reaction of products. This work provides an effective and feasible strategy to increase the efficiency of artificial photosynthesis.

## Abstract (web summary)

The stoichiometric photoreaction of CO_2_ with H_2_O is one of the big challenges in photocatalysis. An artificial photosynthetic system based on a direct and indirect Z-scheme heterostructure is synthesised, enabling simultaneous CO_2_ reduction to HCOOH and H_2_O oxidation to O_2_.

## Introduction

Solar-driven photocatalytic conversion of carbon dioxide (CO_2_) to valuable organics or solar fuel is an attractive solution to both current energy and environment problems^1–4^. Reduction of CO_2_ under visible light accounting for 45% sunlight energy by water rather than organic compound as an electron donor is the ultimate goal of photocatalysis. Enormous efforts have been devoted to developing a highly efficient photocatalyst for this prospect^5–7^. However, so far none of photocatalyst systems is satisfactory. Development of novel photocatalyst or system to realize highly efficient conversion of CO_2_ remains the focus of future research.

In various reduction products of CO_2_, including CO, HCOOH, CH_3_OH, HCHO, CH_4_, etc., HCOOH is a chemical with wide applications^8^, and even is considered as a promising bio-renewable feedstock for fine chemicals^9^. Although HCOOH as a two-electron-transfer product has the lowest degree of reduction among conversion products of CO_2_, all the reported photocatalysts to date still suffer from very low efficiency for HCOOH similar to other organic products^10–13^. This implies that the photocatalytic reduction of CO_2_ to HCOOH is not easier than the reduction to other products. It is well known that the photocatalytic conversion of CO_2_ with H_2_O involves two half-reactions, i.e. the reduction of CO_2_ by the photogenerated electrons and protons, and the oxidation of H_2_O by the photogenerated holes. However, most of the reported photocatalysts could not catalyse simultaneously reduction of CO_2_ to HCOOH and oxidation of H_2_O to O_2_^14^, and only work in the presence of organic hole-scavengers (e.g. triethanolamine (TEOA), trimethylamine (TEA), or ethylenediaminetetraacetic acid (EDTA))^15,16^. Such photocatalytic CO_2_ reduction at the cost of sacrificial electron donors is not sustainable and likely economically unsound. Even using hole scavenger, the photocatalysts only exhibit a formation rate of HCOOH with tens of micromoles, typically such as some metal-organic framework (MOF) materials (NH_2_-MIL-125(Ti), MIL-101(Fe))^17,18^, inorganic–organic hybrid materials (a binuclear ruthenium(II) complex coupled with Ag/C_3_N_4_ (ref. ^19,20^), Cu(I) complex photosensitized Mn(I) complex catalysts^21^), and metal sulfide semiconductors ((Mo−Bi)S_*x*_/CdS)^22^. The C and Fe co-doped LaCoO_3_ was reported to display an HCOOH yield up to 128 μmol g^−1^ h^−1^ without sacrificial reagent, but the oxidation product O_2_ was not analysed^23^. Such photocatalytic CO_2_ reduction without accompanying oxidation half-reaction is inexplicable. The photosynthesis essentially requires the stoichiometric photocatalytic CO_2_ reduction and H_2_O oxidation which remains a great challenge in photocatalysis^24^.

Silicon carbide (SiC), a metal-free semiconductor material, possesses a moderate wide band gap (2.4 eV) with an enough negative CB (ca. −1.1 V) to satisfy multielectron reactions of CO_2_ reduction with H_2_O into carbon fuel and oxygen by solar energy^25,26^. So it has been considered as a promising photocatalyst for CO_2_ conversion since the early research work^27^. However, the expected photocatalytic efficiency has not been achieved so far. This is due to very large difference between electron and hole migration rates in SiC (electron mobility 700 cm^2^ V^−1^ s^−1^, hole mobility 90 cm^2^ V^−1^ s^−1^), which leads to the accumulation of photogenerated holes in the bulk and in turn suppresses the further generation of electrons under light irradiation. This makes the photogenerated carriers to be short-lived, especially the oxidation ability to be poor^28,29^. Moreover, the pristine SiC is lack of active sites for CO_2_ adsorption and activation. Therefore, it is desirable to find the suitable cocatalysts to modify SiC. Additionally, the thermodynamically favourable backward reaction of the produced organics with oxygen on the photocatalyst surface is detrimental to decrease efficiency of CO_2_ with pure H_2_O in the conversional one-pot reaction. These problems can be concurrently solved by constructing the multi-photocatalyst integration systems in which the oxidation and reduction reactions are independent in space but coupled in the transfer of photogenerated charges.

Here, we report a Cu_2_O–Pt/SiC/IrO_*x*_ hybrid photocatalyst, which is prepared by loading the photo-oxidation unit (IrO_*x*_) and the photoreduction unit (Cu_2_O–Pt) on SiC surface. This configuration can enhance the lifetime of photogenerated charges and the CO_2_ adsorption, and thus the photocatalytic efficiency. Furthermore, we construct a spatially separated reaction system consisting of two reaction chambers analogous to the natural photosynthetic systems. One chamber is loaded with the Cu_2_O–Pt/SiC/IrO_*x*_ photocatalyst and Fe^2+^ for CO_2_ reduction, while the other chamber with the known Pt/WO_3_ and Fe^3+^ for H_2_O oxidation, and the two chambers are divided by a Nafion membrane that allows Fe^2+^ and Fe^3+^ ions to permeate through. This design facilitates H_2_O oxidation half-reaction and suppresses the backward reaction of the products. For the photocatalytic reaction of CO_2_ with H_2_O to HCOOH and O_2_, the system shows very high photocatalytic efficiency under visible light irradiation. The HCOOH yield is as high as 896.7 μmol g^−1^ h^−1^ for the long-term reaction, 527 times higher than that of the pristine SiC (1.7 μmol g^−1^ h^−1^) in the conversional one-pot reaction. Most importantly, O_2_ with a stoichiometric ratio is evolved concurrently. To the best of our knowledge, such high activity for reaction of CO_2_ with pure H_2_O to HCOOH and O_2_ is rarely reported before.

## Results and discussion

### Configuration and composition of photocatalysts

**Fig. 1:**
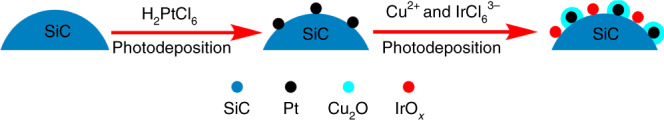
Formation process of Cu_2_O–Pt/SiC/IrO_*x*_ by photodeposition. Schematic representation of Cu_2_O–Pt/SiC/IrO_x_ synthesis via controlled photodeposition.

Figure 1 illustrates the formation process of the photocatalyst Cu_2_O–Pt/SiC/IrO_*x*_ through the step-by-step photodeposition of Pt, Cu_2_O and IrO_*x*_ on 3C-SiC (face centre cubic phase of SiC) surface. First, Pt nanoparticles were loaded onto SiC by a simple photodeposition to obtain Pt/SiC sample. Then the resulting Pt/SiC samples were dispersed in aqueous solution containing both Cu^2+^ and IrCl_6_^3−^ ions with UV light illumination, which led Cu^2+^ to reduction into Cu_2_O species and IrCl_6_^3−^ to oxidation into IrO_*x*_. Due to higher work function (5.6 eV) of Pt, the photogenerated electrons on SiC migrated to Pt and the photogenerated holes remained on SiC^30,31^. Thus the reduction reaction occurred on the Pt particle, while the oxidation reaction did on SiC. We thus concluded that the Cu_2_O was deposited on the Pt, while the IrO_*x*_ on SiC. The resulting Cu_2_O–Pt and IrO_*x*_ were located at different region of SiC surface. For comparison, the two reference samples, Cu_2_O–Pt/SiC and Pt/SiC/IrO_*x*_, were prepared also in the similar conditions with Cu^2+^-contained solution and IrCl_6_^3−^-contained solution, respectively. The loading amounts of cocatalysts on SiC samples were controlled by the photodeposition time (0.5–15 h) and then were quantified by a quadrupole inductively coupled plasma mass spectrometry (ICP-MS), as summarized in Supplementary Table 1 and Supplementary Note 1. The contents of Pt, Cu_2_O and IrO_*x*_ on the samples were controlled in the range of 0.83–2.6, 0.52–2.7 and 0.87–3.2 wt%, respectively. Based on the photocatalytic CO_2_ reduction results, the optimal amount of Pt, Cu_2_O and IrO_*x*_ is ascertained to be 1.3%, 1.8% and 2.2 wt% for Cu_2_O–Pt/SiC/IrO_*x*_ photocatalyst, respectively. For the sake of brevity, hereafter the optimal photocatalyst with 1.3 wt% Pt, 1.8 wt% Cu_2_O and 2.2 wt% IrO_*x*_ is referred simply to Cu_2_O–Pt/SiC/IrO_*x*_, unless specifically noted otherwise.

**Fig. 2:**
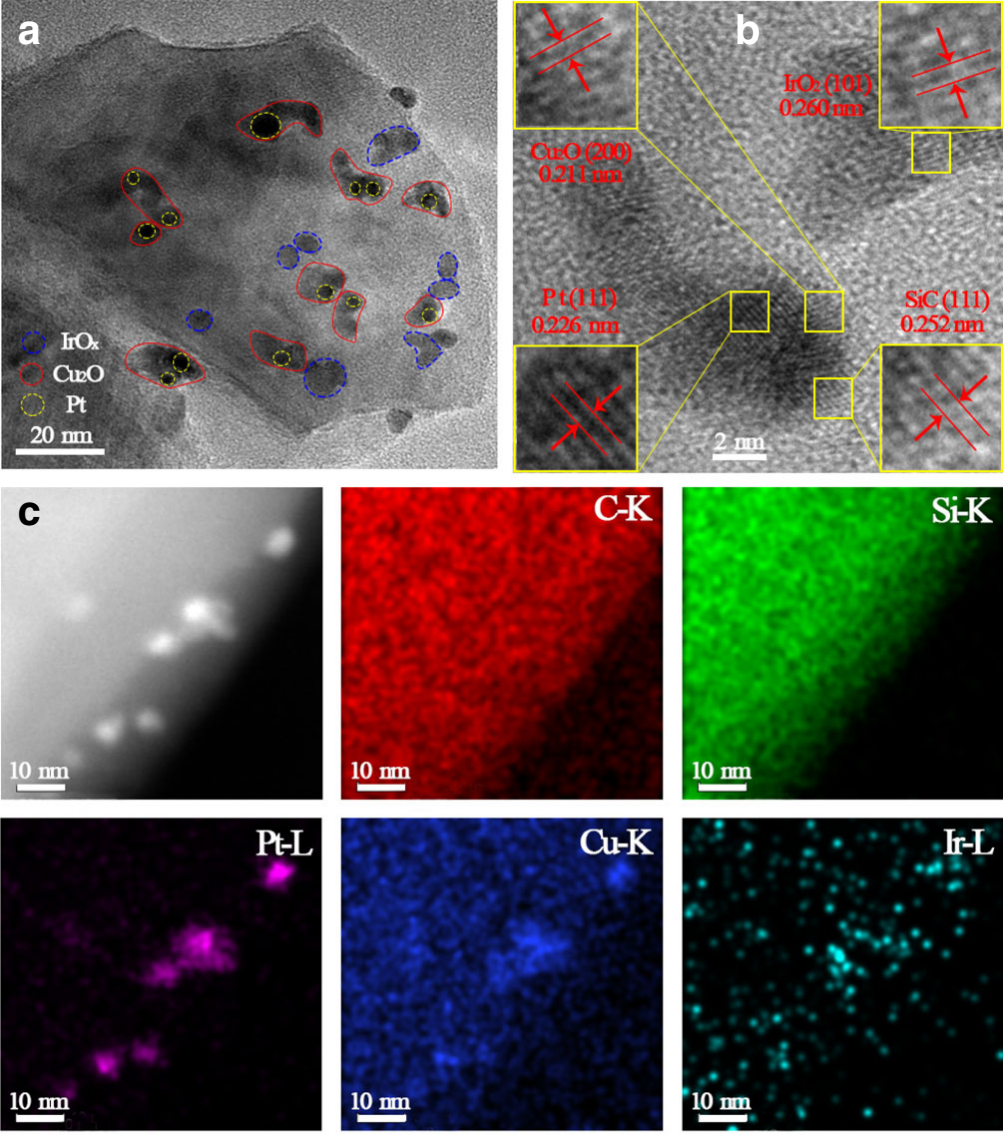
Spatial location of cocatalysts. **a** TEM, **b** HRTEM images and **c** STEM image and corresponding EDS mapping profiles for C-K, Si-K, Pt-L, Cu-K and Ir-L of Cu_2_O–Pt/SiC/IrO_*x*_.

The samples were firstly characterized by X-ray powder diffraction (XRD) (Supplementary Fig. 1, Supplementary Note 2). For all samples, except the highly crystalline cubic phase SiC (JCPDS No. 65-0360), no XRD peaks corresponding to the cocatalysts (Pt, Cu_2_O and IrO_*x*_) are observed due to their low contents and high dispersion on SiC surface. The BET specific surface area of SiC has a slight reduction from ~18 m^2^ g^−1^ for bare SiC to ~14 m^2^ g^−1^ for Cu_2_O–Pt/SiC/IrO_*x*_ (Supplementary Fig. 2, Supplementary Note 3), possibly because the cocatalysts with small-size particles block the micropore structure of SiC. The spatial locations of Pt, Cu_2_O and IrO_*x*_ species on SiC surface were visualized by transmission electron microscopy (TEM) and HRTEM images (Fig. 2). The Pt nanoparticles have a uniform size of ca 2–4 nm and evenly distribute on the surface for all samples, as marked with yellow dotted circle (Fig. 2a, Supplementary Fig. 3). Cu_2_O was deposited over Pt nanoparticles to form a Cu_2_O–Pt intimate contact configuration for both Cu_2_O–Pt/SiC (Supplementary Fig. 3c, Supplementary Note 4) and Cu_2_O–Pt/SiC/IrO_*x*_ samples (Fig. 2a) as marked with red circle, while the IrO_*x*_ species are not deposited at the same location as Cu_2_O–Pt species for both Pt/SiC/IrO_*x*_ (Supplementary Fig. 3d) and Cu_2_O–Pt/SiC/IrO_*x*_ samples (Fig. 2a), as marked with blue dotted circle. The lattice spacings of Cu_2_O–Pt/SiC/IrO_*x*_ samples (Fig. 2b, Supplementary Fig. 3) are 0.252, 0.226, 0.211 and 0.260 nm assigning to the (111) facet of SiC, (111) facet of Pt, (200) facet of Cu_2_O and (101) facet of IrO_2_ (refs. ^32–35^), respectively. The Cu_2_O–Pt intimate contact structure is further testified by STEM-EDS mapping (Fig. 2c, Supplementary Fig. 4). In the selected area, Pt has the same distribution and appears at almost the same position as Cu, further demonstrating the deposition of Cu_2_O over Pt particles. Nevertheless, there is also a part of Cu to be deposited on SiC surface. IrO_*x*_ looks like a random deposition on the entire surface of SiC due to very small cluster particles, but it is separated from Cu_2_O–Pt on the SiC surface, because the distribution of Ir-L in mapping images (Supplementary Fig. 5, Supplementary Note 4, Fig. 2c) has an obvious difference from that of other elements through careful comparison.

**Fig. 3:**
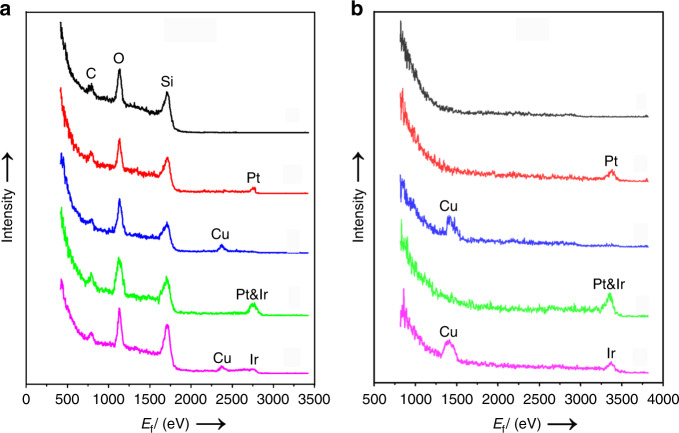
The chemical composition on the outermost surface. HS-LEIS spectra using **a** 3 keV ^4^He^+^ and **b** 5 keV ^20^Ne^+^ for the samples: SiC (black), Pt/SiC (red), Cu_2_O–Pt/SiC (blue), Pt/SiC/IrO_*x*_ (green) and Cu_2_O–Pt/SiC/IrO_x_ (pink).

The chemical composition distribution of the outermost layer on SiC surface was further analysed by a high-sensitivity low-energy ion scattering (HS-LEIS) studies. 3 keV ^4^He^+^ HS-LEIS spectra (Fig. 3a) give the signal of the light elements on the outer surface (such as C, O and Si), but have poor sensitivity to Cu, Pt and Ir heavy elements. Figure 3b shows the 5 keV ^20^Ne^+^ HS-LEIS spectra of SiC, Pt/SiC, Cu_2_O–Pt/SiC, Pt/SiC/IrO_*x*_ and Cu_2_O–Pt/SiC/IrO_*x*_. Pt element is detected on the outmost surface for Pt/SiC. Only Cu element is observed on Cu_2_O–Pt/SiC, clearly indicating that Cu_2_O is deposited on the surface of Pt nanoparticles. Although HS-LEIS peaks of Pt and Ir cannot be resolved using 5 keV ^20^Ne^+^ because their atomic mass is too close, it should be noted that intensity of the fused peaks of Pt and Ir at 3367 eV for Pt/SiC/IrO_*x*_ is significantly stronger than that of sole Pt peak in Pt/SiC. This indicates that both IrO_*x*_ and Pt coexist on the outermost surface of Pt/SiC/IrO_*x*_. However, the peak at 3367 eV for Cu_2_O–Pt/SiC/IrO_*x*_ samples weakens significantly as compared with Pt/SiC/IrO_*x*_. Since Pt is covered by Cu_2_O in Cu_2_O–Pt/SiC/IrO_*x*_, the low peak at 3367 eV of Cu_2_O–Pt/SiC/IrO_*x*_ can be only assigned to IrO_*x*_ on the outermost surface. Therefore, the HS-LEIS results constitute another strong evidence that the Cu_2_O–Pt and IrO_*x*_ cocatalysts are spatially separated on SiC surface for Cu_2_O–Pt/SiC/IrO_*x*_.

**Fig. 4:**
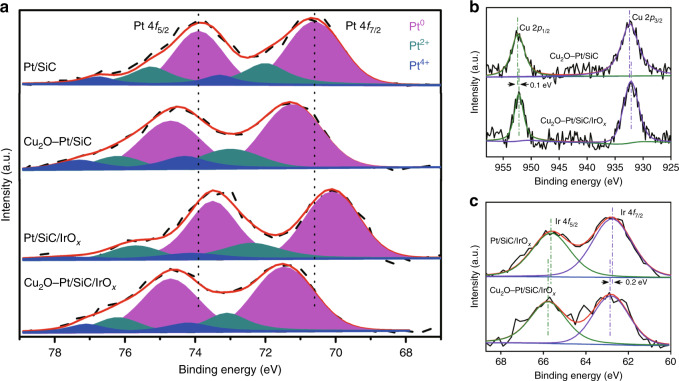
The chemical states of cocatalysts. **a** Pt 4*f* XPS spectra, **b** Cu 2*p* XPS spectra, and **c** Ir 4*f* XPS spectra of Pt/SiC, Cu_2_O–Pt/SiC, Pt/SiC/IrO_*x*_ and Cu_2_O–Pt/SiC/IrO_*x*_ samples.

The chemical states of the Pt, Cu and Ir elements in samples were analysed by XPS (Fig. 4). Three sets of Pt 4*f* XPS peaks can be assigned to metallic Pt^0^ and partially oxidized Pt^2+^ and Pt^4+^ species^36^. The ratio of Pt^0^ is calculated to be accounting for 70 ± 4% of the sum Pt species for the all Pt-contained samples (Supplementary Table 2). Cu species and Ir species are mainly in the state of Cu_2_O and mixed valence oxides (IrO_*x*_), respectively^37,38^. Nevertheless, the binding energies (BE) of Pt, Cu or Ir have obvious differences among samples. For Pt/SiC, the BE of Pt 4*f*_7/2_ and Pt 4*f*_5/2_ are respectively 70.8 and 74.1 eV, which are lower than that of the pure metallic Pt (Pt 4*f*_7/2_ = 71.2 eV)^39^. This is because the electron transfer occurs from the SiC substrate to Pt particles with higher work function^40,41^. When Cu_2_O is subsequently deposited on Pt/SiC to form Cu_2_O–Pt/SiC, the BE of Pt 4*f*_7/2_ shifts towards higher energy (71.1 eV), but is still slight lower than that of metallic Pt, indicating the electron transfer still from SiC to Cu_2_O under mediation of Pt nanoparticles. On the contrary, when IrO_*x*_ is deposited onto Pt/SiC to form Pt/SiC/IrO_*x*_, the BE of Pt 4*f* shifts to lower position. This indicates that IrO_*x*_ deposition induces the electron transfer from IrO_*x*_ to the SiC surface and thus enhances the electron transfer to Pt particles. In the case of the co-deposition of IrO_*x*_ and Cu_2_O on Pt/SiC, the BE of Pt 4*f* in Cu_2_O–Pt/SiC/IrO_*x*_ is comparable with Cu_2_O–Pt/SiC. This demonstrates that the electrons are finally transferred from both SiC and IrO_*x*_ into Cu_2_O. As a result, the BE of Cu 2*p* for Cu_2_O–Pt/SiC/IrO_*x*_ is also lower than that of Cu_2_O–Pt/SiC. In reverse, the BE of Ir 4*f* for Cu_2_O–Pt/SiC/IrO_*x*_ is slightly positively shifted as compared with Pt/SiC/IrO_*x*_. The above results show the existence of the strong interfacial interaction between co-catalyst and SiC, which would be favourable for the electron migration and transfer in Cu_2_O–Pt/SiC/IrO_*x*_.

### Photocatalytic performance of CO_2_ reduction with H_2_O

**Fig. 5:**
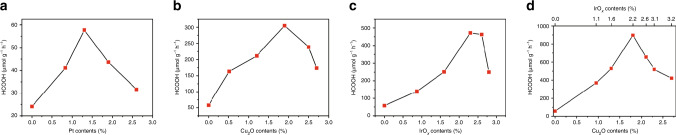
The optimal contents of cocatalysts for CO_2_ reduction. Change in HCOOH evolution rate **a** on Pt/SiC with increasing Pt content, **b** on Cu_2_O–Pt (1.3 wt%)/SiC with increasing Cu_2_O, **c** on Pt (1.3 wt%)/SiC/IrO_*x*_ with increasing IrO_*x*_, and **d** on Cu_2_O–Pt/SiC/IrO_*x*_ with increasing Cu_2_O and IrO_*x*_, in spatially separated reactor under visible light irradiation. Reaction conditions: 50 mg SiC-based photocatalyst and 300 mL of 2 mM FeCl_2_ aqueous solution were placed in the CO_2_-reduction chamber, 100 mg Pt/WO_3_ and 300 mL of 2 mM FeCl_3_ aqueous solution in the H_2_O oxidation chamber, the pH of solution was adjusted to 2.3 by adding hydrochloric acid to prevent precipitation of the iron ions aqueous solution.

The photocatalytic CO_2_ reduction was performed in the spatially separated reaction system. Fe^2+^ and Fe^3+^ were added respectively in the CO_2_-reduction compartment and the H_2_O-oxidation compartment in the beginning of the reaction (see Supplementary Fig. 6)^42,43^. During the photoreaction process, Fe^3+^ and Fe^2+^ ions are able to permeate through the Nafion membrane driven by the concentration gradient. The photocatalytic activities of the samples for the reaction of CO_2_ with H_2_O were tested under visible light irradiation in order to find out the optimal contents of cocatalysts, as shown in Fig. 5 and Supplementary Table 1. HCOOH is detected as a main product for all photocatalysts. The bare SiC only shows 24.1 μmol g^−1^ h^−1^ of HCOOH yield. Over Pt/SiC samples, the HCOOH yield shows a volcanic curve with increasing Pt contents and the highest HCOOH yield (57.7 μmol g^−1^ h^−1^) occurs at 1.3 wt% Pt content (Fig. 5a). On the Cu_2_O–Pt/SiC sample with 1.3 wt% Pt, the product HCOOH shows the highest yield (304.6 μmol g^−1^ h^−1^) at 1.8 wt% content of Cu_2_O (Fig. 5b). On the Pt/SiC/IrO_*x*_ with the optimum Pt content (1.3 wt%), the optimal loading content of IrO_*x*_ is 2.2 wt% at which the HCOOH yield is ca. 472.0 μmol g^−1^ h^−1^ (Fig. 5c). When both IrO_*x*_ and Cu_2_O are simultaneously deposited on the optimal Pt/SiC, the highest yield of HCOOH, 896.7 μmol g^−1^ h^−1^, occurs at ca. 2.2 wt% of IrO_*x*_ and 1.8 wt% of Cu_2_O. When Cu_2_O content is higher than 1.8 wt%, further increasing Cu_2_O photodeposition induces more amount of Cu_2_O on both Pt particles and the exposed SiC surface. The deposited Cu_2_O on SiC could block the optical absorption of SiC, and an over-thick Cu_2_O layer on Pt surface is also unfavourable to CO_2_ reduction on Cu_2_O–Pt^32^. The higher IrO_*x*_ contents <2.2 wt%, the more active sites of IrO_*x*_ are provided to enhance the Fe^2+^ oxidation. However, an excess amount of IrO_*x*_ may lead to the growth of IrO_*x*_ into large particles on SiC surface, which is harmful to the photocatalytic reaction. The HCOOH yield over the optimal Cu_2_O–Pt/SiC/IrO_*x*_ is almost 37 times of the activity of the bare SiC. To the best of our knowledge, the photocatalytic efficiency of our system is substantially higher than that of other various photocatalysts reported so far (seen activity comparison in Supplementary Table 3).

**Fig. 6:**
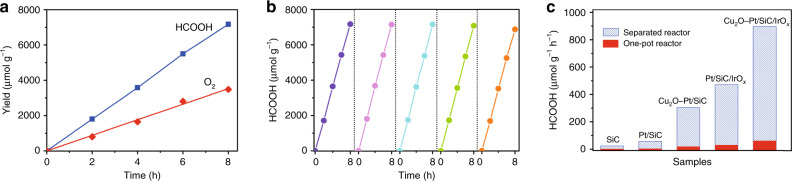
Photocatalytic performance of CO_2_ reduction with H_2_O. **a** Evolutions of HCOOH and O_2_ as a function of illumination time and **b** cycle experiment of HCOOH evolution in the spatially separated reaction system with Cu_2_O–Pt/SiC/IrO_*x*_ as the reduction side photocatalyst. **c** Comparison for the HCOOH evolution between in the spatially separated reaction system and in the conventional one-pot reactor with different samples as the reduction side photocatalyst.

O_2_ is confirmed as main product evolving in the Pt/WO_3_ oxidation chamber of the separated reaction system. Figure 6a shows the production of O_2_ in the Pt/WO_3_ chamber and HCOOH in the Cu_2_O–Pt/SiC/IrO_*x*_ chamber vs. irradiation time. Both yields of HCOOH and O_2_ increase linearly, and reach 7200 and 3300 μmol g^−1^ during 8 h photocatalytic reactions, respectively. Moreover, the ratio of product O_2_ to HCOOH is close to the stoichiometric number of reaction (2CO_2_ + 2H_2_O → 2HCOOH + O_2_) in the whole process. Influence of the Pt amount of Pt/WO_3_ on the photocatalytic O_2_ and HCOOH evolutions in the separated reaction system was also investigated (Supplementary Fig. 7). As decreasing Pt content to 0.5 wt% of Pt/WO_3_, the O_2_ and HCOOH yields decrease to 296.6 and 618.7 μmol g^−1^ h^−1^, respectively. However, the ratio of O_2_ to HCOOH is still close to the stoichiometric number. When the Pt content increases to 1.5 wt%, both O_2_ and HCOOH evolution is almost the same as that of 1.0 wt% Pt. The 1.0 wt% Pt content is optimal for the Pt/WO_3_ in the separated reaction system. Obviously, Pt content of Pt/WO_3_ does not affect the stoichiometric ratio of product HCOOH to O_2_. This is because Fe^3+^/Fe^2+^ redox couple acts as the electron transfer medium between CO_2_ reduction and H_2_O oxidation by the concentration diffusion through the Nafion membrane, which makes the reaction stoichiometrically proceeded in the reaction process. However, the HCOOH yield shows a downtrend as prolonging the reaction time (Supplementary Fig. 8). The decrease of activity is because pH value of the reaction solution is increased from 2.3 at the initial stage to 4.5 after 12 h reaction along with the consumption of H^+^ in the process of CO_2_ reduction. The increase in solution alkalinity can result in hydrolysis of Fe^3+^ to FeOOH or Fe(OH)_3_, as confirmed by the Fe 2*p* XPS spectrum of the sample after the reaction (Supplementary Fig. 9). However, the activity is highly stable in five cycles of total 40 h (Fig. 6b) when the Cu_2_O–Pt/SiC/IrO_*x*_ is centrifuged out and then added to the fresh solution before each new cycle. Therefore, the increase in solution alkalinity during the photocatalytic reaction is one of reasons effecting stability of the reaction, which should be avoided by increasing solution acidity.

The Cu_2_O–Pt/SiC/IrO_*x*_ samples before and after the photocatalytic reaction were characterized by XPS (Supplementary Fig. 10), indicating some differences in chemical states of Pt, Ir and Cu. However, the changes of metal states are likely to have no significantly influence or be not main factor on the photocatalytic activity stability. (i) For Pt, the XPS Pt 4*f* peaks narrow down and the shoulder peaks become weak for the used sample, but the BE values of main peaks keep almost unchanged before and after the photocatalytic reaction. The main peaks at the BEs of 74.7 eV (Pt 4*f* 5/2) and 71.5 eV (Pt 4*f* 7/2) are attributed to Pt(0). The shoulder peaks are belonged to Pt^2+^ and Pt^4+^ species. It can be seen that a part of high valence Pt species were translated also into Pt (0) after longtime reaction, which could be beneficial to the photocatalysis. (ii) For IrO_*x*_, the Ir 4*f* peaks not only become narrow but also shift towards lower energy after the photocatalytic reaction. The wide Ir 4*f* peaks of both the fresh and the used samples cover Ir^0^, Ir^3+^ and Ir^4+^ species, indicating the mix valence state feature of IrO_*x*_. Accordingly, the Ir 4*f*_7/2_ peak can be deconvoluted into a contribution of Ir^0^ (61.3 eV), Ir^4+^ (62.4 eV) and Ir^3+^ (63.5 eV) species^44–46^. The phenomena are universal for Ir-loaded catalysts, which is also the reason why iridium oxide is usually be expressed as IrO_*x*_ rather than IrO_2_^47^. It is estimated from the peak intensities that the contents of Ir^4+^ are increased, while Ir^3+^ contents are decreased in the used sample as compared with the fresh sample (see Supplementary Table 4). Such a change in the Ir 4*f* XPS spectra can be explained by the changes in the crystallinity and coordination numbers^48,49^. For the fresh sample, the broader and higher BE peaks suggest the existence of partial amorphous or high oxygen coordinated Ir species. For the used sample, the shift of BE of Ir 4*f* peak towards lower energy indicates an increase in the rutile phase IrO_2_ during the photocatalytic process since the IrO_*x*_ could be mainly excited from the d(t_2g_) to the d(e_g_) band (1.5–2.75 eV) under visible light irradiation based on the literature^50^. It is possible that the change in Ir valence state or crystallinity do not affect the photoinduced d–d transition and thus photocatalytic performance. (iii) As for Cu_2_O, the Cu 2*p* XPS spectrum shows a minor change. The BEs of the main peaks of Cu 2*p* remain almost the same before and after the reaction, only a minuscule shift towards lower energy. We could not exclude the possibility that a small amount of Cu(I) was translated into Cu(0) after the photocatalytic reaction. Because some Cu_2_O is deposited synchronously on the SiC surface, the small change of Cu valence state can occur partly for these Cu_2_O species, which could have no remarkable influence on the photocatalytic performance.

Other two control experiments were carried out also. When ^13^CO_2_ instead of CO_2_ was used as a reactant, ^13^C NMR analysis for the reaction solution verifies that only a strong peak at 171.5 ppm attributed to the ^13^C in H^13^COOH was observed (Supplementary Fig. 11)^51^. When CO_2_ was not added in the reaction system, no HCOOH was detected. These results show that the product HCOOH is formed from the photocatalytic CO_2_ reduction. As H_2_^18^O instead of H_2_O is used as the reactant in a small dose simulated reaction, the mass spectroscopy analysis gives a main peak at *m*/*z* = 36 corresponding to ^18^O_2_ (Supplementary Fig. 12), confirming the product O_2_ originating from H_2_O oxidation. Careful analysis reveals that except HCOOH no carbon-containing products, such as CO, HCHO and CH_4_, come into being in the gas phase. Nevertheless, there is only a very slight amount of H_2_ to evolve as a by-product (Supplementary Fig. 13), implying existence of competition between the reduction of H^+^ to H_2_ and the reduction of CO_2_ to HCOOH by photogenerated electrons. In the absence of CO_2_ atmosphere, however, only a slightly enhanced amount of H_2_ evolves in the gas phase (Supplementary Fig. 14), although the photocatalytic reaction is performed in the acidic aqueous solution. This indicates that the SiC-based photocatalyst is not good for H_2_ evolution from water, which may be because that it lacks the active sites or has high overpotential for the H_2_ evolution. However, for the reduction of CO_2_, the SiC-based photocatalyst can be particularly effective.

The photocatalytic activity for the reaction of CO_2_ with H_2_O was also evaluated in the conventional one-pot reactor, where we added simultaneously SiC-based catalyst, Pt/WO_3_ and Fe^2+^/Fe^3+^. HCOOH and O_2_ are also detected as main products. Figure 6c compares the HCOOH yields under two different reaction modes (see also Supplementary Table 5). It can be seen that all the samples show much higher HCOOH yield with the spatially separated reaction system than with the one-pot reaction mode. For SiC, Pt/SiC, Cu_2_O–Pt/SiC, Pt/SiC/IrO_*x*_ and Cu_2_O–Pt/SiC/IrO_*x*_ photocatalyst, the HCOOH yield is 1.7, 3.5, 18.6, 30.4 and 61.5 μmol g^−1^ h^−1^ with the one-pot reaction, while it is 24.1, 57.7, 304.6, 472.0 and 896.6 μmol g^−1^ h^−1^ with the spatially separated system, respectively. Obviously, the photocatalytic activity of the spatially separated system is ca *~*15 times higher than that in the one-pot reaction reactor for each photocatalyst. In the one-pot reaction system, the backward reaction of HCOOH re-oxidization by O_2_ should be one of the reasons for the low evolution of HCOOH and O_2_. Both Fe^3+^ competing with CO_2_ for the generated electrons and Fe^2+^ competing with H_2_O for the photogenerated holes could also take place at the same time. However, the effects of the later could be weaker than that of the former, because the evolutions of HCOOH and O_2_ are lower if no adding Fe^3+^ and Fe^2+^ (see the first column in Supplementary Table 5). This could be one of reasons for high efficiency of the spatially separated system. However, the reasonableness of this inference requires the following two premises: the product HCOOH (i) does not diffuse to the oxygen evolution chamber from the reduction chamber through the Nafion membrane and (ii) is not oxidized in the reduction chamber by the Fe^3+^, with increase in the production of HCOOH. Accordingly, two additional experiments were done. The permeation of HCOOH across the Nafion membrane from the HCOOH solution (1200 μmol L^−1^, corresponding to the maximum yield of HCOOH in the separated system for 8 h reaction) to pure H_2_O is determined firstly. Only very limited amount of HCOOH (<5%) is diffused to the pure water across the Nafion membrane within 8 h (Supplementary Fig. 15). Another experiment is the solution (pH = 2.3) with HCOOH (1200 μmol L^−1^) and Fe^2+^/Fe^3+^ (2 mmol L^−1^) under visible light illumination. The concentration of HCOOH remains almost unchanged (Supplementary Fig. 16). These indicate that our new system is indeed effective to prevent the backward reaction. The wavelength-dependent evolution of HCOOH was also performed to gain the apparent quantum yield (AQY) (Supplementary Fig. 17). Obviously, the AQY of HCOOH evolution on the optimal Cu_2_O–Pt/SiC/IrO_*x*_ sample is well matched with the optical absorption spectra. Under 400 nm light irradiation, the AQY of HCOOH evolution can be reached near 1.44%.

### The in situ CO_2_ adsorption FT-IR spectra

**Fig. 7:**
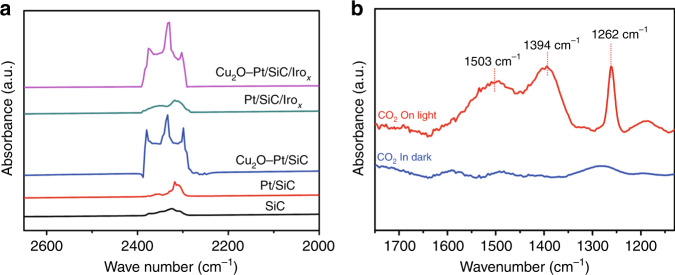
Adsorption of CO_2_ on photocatalysts. **a** In situ FT-IR spectra of CO_2_ adsorbed on different photocatalysts. **b** In situ FT-IR spectra of CO_2_ adsorbed on Cu_2_O–Pt/SiC/IrO_*x*_ before and after visible light irradiation. All the spectra are the difference spectra between after and before CO_2_ adsorption.

The photocatalytic activity of Cu_2_O–Pt/SiC/IrO_*x*_ is always higher than that of the other samples both in the spatially separated system and in the one-pot reactor. The HCOOH yield on the optimal Cu_2_O–Pt/SiC/IrO_*x*_ sample in the spatially separated system is 37 and 527 times higher than that on the bare SiC in the spatially separated system and in the one-pot reactor, respectively. Obviously, the high efficiency of Cu_2_O–Pt/SiC/IrO_*x*_ for the reaction of CO_2_ with H_2_O to HCOOH can be related to the photocatalyst surface feature. The in situ CO_2_ adsorption FT-IR spectra were measured to gain insight into the effect of surface feature. As shown in Fig. 7a, all photocatalysts show the multiple IR adsorption peaks of CO_2_ in the range of 2200–2500 cm^−1^ in the dark. It is noteworthy that both the samples containing Cu, i.e. Cu_2_O–Pt/SiC and Cu_2_O–Pt/SiC/IrO_*x*_, show much stronger CO_2_ adsorption band than nude SiC, Pt/SiC and Pt/SiC/IrO_*x*_. This implies that CO_2_ molecules are mainly adsorbed on the Cu_2_O co-catalyst in Cu_2_O–Pt/SiC and Cu_2_O–Pt/SiC/IrO_*x*_^32,52^. Figure 7b shows the in situ CO_2_ adsorption FT-IR spectra on Cu_2_O–Pt/SiC/IrO_*x*_ in the range of 1700–1200 cm^−1^ after and before light irradiation, which is a reflection of chemical adsorption. No absorption attributable to C–O species appears on Cu_2_O–Pt/SiC/IrO_*x*_ in the absence of CO_2_ gas (omitted). As introduction of CO_2_, Cu_2_O–Pt/SiC/IrO_*x*_ shows very weak several absorption peaks before visible light irradiation. However, three strong absorption bands appear upon visible light irradiation. Both the wide absorption band at 1394 cm^−1^ and the weak band at 1503 cm^−1^ are ascribed to bidentate carbonate species bonded to the Cu_2_O surface, while the absorption band at 1262 cm^−1^ is assigned to a monodentate carbonate to the Cu_2_O surface^53,54^. This demonstrates that CO_2_ molecules are activated at room temperature by the Cu_2_O co-catalyst on Cu_2_O–Pt/SiC/IrO_*x*_ surface under light irradiation. Moreover, the acidic aqueous solution (pH = 2.3) is helpful for formation of the carboxyl radical intermediate (·COOH) or the formate anion (HCOO^−^) that is further easily converted into HCOOH^14,55^. This could be the second possible reason for the high efficiency of the spatially separated system. High selectivity of product HCOOH may be related to the different reaction mechanism dependent on the reaction conditions, such as the photocatalyst states (defects, crystal faces, doping, etc.), reaction conditions (temperature, pH, CO_2_ concentration, reactant phase, etc.), co-catalyst and so on. In the gas (CO_2_, H_2_O vapour)–solid (catalyst.) mode, it has been found that the SiC-based composite (MoS_2_/SiC) photocatalyzed the CO_2_ reduction into CH_4_ undergoing HCOOH, HCHO and CH_3_OH intermediates on SiC surface by the hydrogenation pathways^26^. In the present work, the reaction is conducted in gas (CO_2_)–liquid (H_2_O)–solid(catalyst) mode and the acidic aqueous solution. In addition, the reduction of CO_2_ occurs on the Cu_2_O sites rather than SiC, which is helpful for formation of the carboxyl (hydroxyformyl) radical intermediate **·**COOH and HCOOH^14,56^.

### Photoelectrochemical properties of Cu_2_O–Pt/SiC/IrO_*x*_

**Fig. 8:**
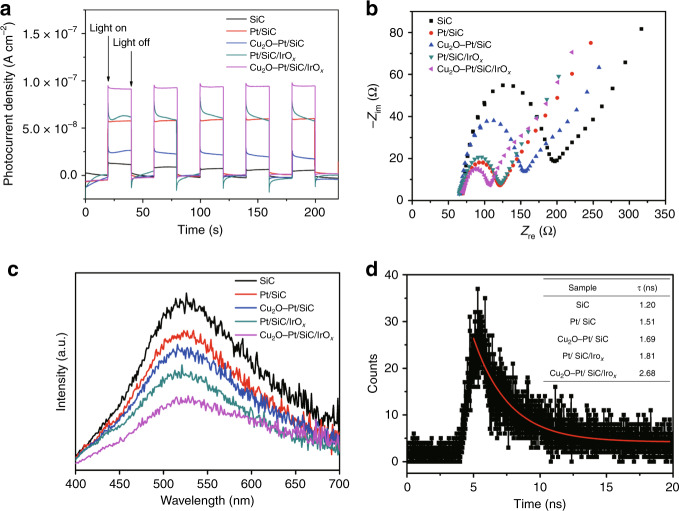
Separation efficiency and lifetime of carriers. **a** Periodic on/off photocurrent response, **b** AC Impedance and **c** PL (photoluminescence) spectra for different photocatalysts. **d** Time-resolved photoluminescence spectroscopy of Cu_2_O–Pt/SiC/IrO_*x*_.

The photoelectrochemical responses and the photoluminescence spectra of these photocatalysts were measured. Figure 8a, b shows the photocurrents and AC impedance of the different samples. The photocurrents increase in order of SiC, Cu_2_O–Pt/SiC, Pt/SiC, Pt/SiC/IrO_*x*_ and Cu_2_O–Pt/SiC/IrO_*x*_, and the AC impedance values decrease in the same order. Obviously, Cu_2_O–Pt/SiC/IrO_*x*_ shows the biggest photocurrent and the smallest AC impedance, indicating the highest electron transfer rate and separation efficiency. However, the sample Cu_2_O–Pt/SiC and Pt/SiC show a slight abnormality, i.e. both the photocurrent increase order and the AC impedance decrease order are inconsistent with the increase order of photocatalytic activity. This can be explained by the dependence of photocatalytic activity on not only the transfer rate and separation efficiency of photogenerated charges but also the surface chemistry characteristics of photocatalyst. Figure 8c is the steady-state photoluminescence (PL) spectra of these samples. The PL intensities decrease in accordance with the order of SiC, Pt/SiC, Cu_2_O–Pt/SiC, Pt/SiC/IrO_*x*_ and Cu_2_O–Pt/SiC/IrO_*x*_, which agree with the change in the photocatalytic activity. Since low PL intensity corresponds to low recombination rate of photogenerated charges, the lowest PL intensity indicates the smallest recombination rate of photogenerated charge for Cu_2_O–Pt/SiC/IrO_*x*_. Figure 8d shows the time-resolved photoluminescence spectroscopy of these samples (see detailed Supplementary Fig. 18), and the corresponding average lifetimes (*τ*) of charge carriers (the inset table). The *τ* value of nude SiC is about 1.2 ns, consistent with the reported literature^57^. Loading Pt, Cu_2_O and IrO_*x*_ nanoparticles lead to an increase in the lifetime *τ*. In these samples, Cu_2_O–Pt/SiC/IrO_*x*_ has the highest *τ* value. The increase in the lifetime *τ* of the charge carriers could increase the probability of their involvement in photocatalytic reactions before recombination^58–60^. So, increases in the transfer rate, separation efficiency and lifetime of photogenerated charges could be the third reason for high efficiency of the spatially separated system.

### Photocatalytic mechanism of the spatially separated system

**Fig. 9:**
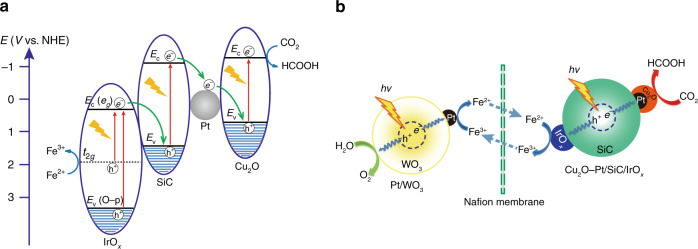
Electronic structure and photocatalytic mechanism. **a** The electron transfer processes in Cu_2_O–Pt/SiC/IrO_*x*_ under light illumination. **b** The proposed mechanism of the separated system for the efficient CO_2_ reduction and O_2_ evolution.

The next issue is the electron structure of Cu_2_O–Pt/SiC/IrO_*x*_ and the photocatalytic mechanism of the spatially separated system. The electron structure parameters of SiC and Cu_2_O were reckoned by combination of the UV–Vis diffuse reflection spectra with the Mott-Schottky analysis (Supplementary Fig. 19). The optical absorption edge of the nude SiC is at 501 nm. The band gap energy and the conduction band (CB) potential of SiC are ca. 2.48 eV and −1.08 V (vs. SHE), respectively. The parent Cu_2_O is estimated to have a band gap of 1.98 eV and CB of −1.28 V. It has been reported that the IrO_*x*_ could be excited from the d(t_2g_) to the d(e_g_) band (1.5–2.75 eV) by visible irradiation and from the O-p band to the d(e_g_) (>3.0 eV) band by ultraviolet irradiation, and its CB is +0.35 V^50^. For the Cu_2_O–Pt/SiC/IrO_*x*_ photocatalyst, Cu_2_O–Pt and IrO_*x*_ cocatalysts are separated each other on SiC surface, and Pt is sandwiched between Cu_2_O and SiC. Thus, Cu_2_O–Pt/SiC/IrO_*x*_ is suggested to have the energy band alignment in Fig. 9a.

If the Cu_2_O co-catalyst directly contacts with SiC without Pt, the photoelectron transfers on the resulting Cu_2_O/SiC composite is speculated to follow the Z-scheme transfer from SiC to Cu_2_O, which has been reported in many literatures^61–63^. Such Z-scheme photoelectron transfer was verified by a photocatalytic probe reaction. When the water solution containing the Cu_2_O/SiC sample and H_2_PtCl_6_ was illuminated by UV light, the reduction reaction (PtCl_6_^2−^ + 4*e*^−^ → Pt + 6Cl^−^) would occur on the Cu_2_O/SiC surface. The TEM image clearly indicates preponderant photodeposition of Pt particles over Cu_2_O particle rather than over SiC surface (Supplementary Fig. 20), unambiguously verifying the Z-scheme electron transfer, i.e. the transfer of photogenerated electron from the CB of SiC to the VB of Cu_2_O. For our Cu_2_O–Pt/SiC/IrO_*x*_ sample, we can reasonably conclude that the Z-scheme electron transfers from the CB of SiC to the valence band (VB) of Cu_2_O would be accelerated by the Pt nanoparticles located between Cu_2_O and SiC, due to the excellent conductivity and high work function of Pt. The controlled experiment shows that the Cu_2_O/SiC displays the HCOOH evolution of about 40.5 μmol g^−1^ h^−1^, which is little higher than that of the pristine SiC but much lower than that of Cu_2_O–Pt/SiC samples (Supplementary Fig. 21). This means that contribution of the Cu_2_O deposited on SiC surface to the activity is very small, and the high activity is mainly due to the embedding of Pt in the interface between Cu_2_O and SiC. The photocatalytic CO_2_ reduction performances of Cu_2_O/SiC/IrO_*x*_ and Pt–Cu_2_O/SiC/IrO_*x*_ were also compared with that of Cu_2_O–Pt/SiC/IrO_*x*_ (Supplementary Fig. 21). Much lower activities of Cu_2_O/SiC/IrO_*x*_ and Pt–Cu_2_O/SiC/IrO_*x*_ than that of Cu_2_O–Pt/SiC/IrO_*x*_ indicates likewise that the Pt sandwiched between the interface of Cu_2_O and SiC is more beneficial to the transfer of photogenerated electrons from SiC to Cu_2_O thus enhances CO_2_ reduction. Simultaneously, another direct Z-scheme photoelectron transfer occurs at the interface between IrO_*x*_ and SiC, because SiC has much more positive VB than CB of IrO_*x*_. As a result, SiC, Cu_2_O and IrO_*x*_ in Cu_2_O–Pt/SiC/IrO_*x*_ all are excited by visible light, the photogenerated electrons from the CB of IrO_*x*_ (+0.35 V) would transfer towards the VB of SiC (+1.40 V). Synchronously, the photogenerated electrons from the CB of SiC (−1.08 V) would transfer towards Pt nanoparticles, and then towards the VB of Cu_2_O (+0.70 V) where they combine with the photogenerated holes at Cu_2_O. On the whole, the coupled direct Z-scheme processes result in the photogenerated electrons accumulating in the CB of Cu_2_O (−1.28 V) where the adsorbed CO_2_ is reduced into HCOOH [*E*(CO_2_/HCOOH) = −0.61 V], while the photogenerated holes on the VB of IrO_*x*_ (t_2g_ = +1.85 V) to oxidize Fe^2+^ into Fe^3+^ [*E*(Fe^2+^/Fe^3+^)  = +0.77 V]^64,65^. Meanwhile, in the H_2_O oxidation chamber, Pt/WO_3_ is excited also by visible irradiation. The photogenerated electrons would transfer from the CB of WO_3_ (+0.74 V) towards Pt and then reduce Fe^3+^ into Fe^2+^ [*E*(Fe^2+^/Fe^3+^) = +0.77 V], while the photogenerated holes of WO_3_ (+2.06 V) would oxidize H_2_O to O_2_ (*E* = +1.23 V). As shown in Fig. 9b, the overall photocatalytic system follows indirect Z-scheme mechanism similar to the natural photosynthesis, i.e. the photocatalytic CO_2_ reduction half-reaction and the photocatalytic H_2_O oxidation half-reaction take place at two separated reactors by the relaying role of Fe^3+^/Fe^2+^ redox couple. To prove that the total reaction is a combination of the spatially separated reduction and oxidation, two controlled experiments were done. When the water solution containing photocatalyst Pt/WO_3_ and Fe^3+^ is illuminated with visible light, O_2_ is generated also as a main product (Supplementary Fig. 22a), validating the oxidation half-reaction in the left side of Fig. 9b. When the solution containing H_2_O, Cu_2_O–Pt/SiC/IrO_*x*_, Fe^2+^ and CO_2_ is illuminated with visible light, HCOOH is detected also as main product (Supplementary Fig. 22b), validating the reduction half-reaction in the right side of Fig. 9b. Therefore, Fe^3+^/Fe^2+^ redox couple makes the reduction of CO_2_ to HCOOH on Cu_2_O–Pt/SiC/IrO_*x*_ and the oxidation of H_2_O to O_2_ on Pt/WO_3_ integrated into the one system like the natural photosynthetic systems. It must be mentioned that the usage mode of Fe^3+^ and Fe^2+^ has little impact on the reaction efficiency. When the Fe^2+^/Fe^3+^ mixed solution was used in both cells of the separated reaction system, HCOOH was produced steadily with the reaction time (Supplementary Fig. 23), and its generation rate is 629.8 μmol g^−1^ h^−1^. When Fe^2+^ and Fe^3+^ were separately loaded in the CO_2_-reduction compartment and the H_2_O-oxidation compartment, the initial generation rate of HCOOH is 896.6 μmol g^−1^ h^−1^. Obviously, the separate addition of Fe^2+^ solution and Fe^3+^ solution in both compartments is more efficient than the loading of Fe^2+^/Fe^3+^ mixed solution in both compartments for the photocatalytic reaction in the separated reaction system. But the separated use mode of Fe^2+^ and Fe^3+^ can only improve initiate photoreaction efficiency. When the redox reaction reaches dynamic steady state, the concentrations of Fe^2+^ and Fe^3+^ also reach a constant gradient in both sides to maintain counter diffusion. Fe^2+^ and Fe^3+^ are not evenly dispersed in either cell according to previous study due to osmosis of Fe ions through Nafion membrane^66^. Even though the concentration of Fe^2+^ and Fe^3+^ is equal in the reaction compartments, the H_2_O oxidation and CO_2_ reduction can proceed over the photocatalysts due to the different adsorption property of Fe^2+^/Fe^3+^ on photocatalyst surface. For example, Fe^3+^ is more favourable to adsorb on WO_3_ surface than Fe^2+^ (ref. ^67^).

## Methods

### Materials

SiO_2_ and H_2_PtCl_6_·6H_2_O were acquired from Aladdin, and the Na_3_IrCl_6_ was supplied from Alfa Aesar. Other reagents used in this work, including glucose, methanol, WO_3_, NaOH, CuSO_4_·5H_2_O and NaIO_3_ were of analytical reagent grade and obtained from Sinopharm Chemical Reagent Co., Ltd. All of the above chemicals were used without further purification.

### Preparation of Pt/SiC photocatalyst

The SiC nanoparticles were prepared by the carbothermic reduction method. Powders of SiO_2_ and glucose were mixed in the molar ratio of Si:C = 1:6 and pulverized in a mortar to well disperse the mixed powders. The well-mixed powder was calcined under Ar atmosphere at 1450 °C for 5 h at a rate of 2 °C min^−1^ in a tubular furnace. The calcined powder was then cooled to room temperature and further purified to remove unreacted raw materials based on our previous report^26^. Briefly, the sample was calcined under O_2_ atmosphere at 873 K for 5 h and steeped with 10 wt% sodium hydroxide solution in order to remove free carbon and unreacted SiO_2_.

Platinum (Pt) was loaded on SiC by the photodeposition method as described in the following steps. The H_2_PtCl_6_ solution with a concentration of 1 mmol L^−1^ was mixed with SiC powder in a quartz cell. In the process of stirring, 2 mL of methanol was added into the mixture. After evacuation, the suspension was irradiated with a 125 W Hg lamp to load Pt nanoparticles onto the SiC. After a certain irradiation time, the obtained sample was washed thoroughly with deionized water and dried in vacuum for 1 h. The irradiation time was changed from 0.5 to 2 h to tune the content of Pt in sample, which was denoted as Pt−*x*h/SiC (*x* = 0.5, 1, 1.5, 2, representing the irradiation time).

### Preparation of Cu_2_O–Pt/SiC and Pt/SiC/IrO_*x*_ photocatalysts

Cu_2_O–Pt/SiC and Pt/SiC/IrO_*x*_ photocatalysts were also prepared by the similar procedure by using Pt/SiC (Pt-1h/SiC) instead of pure SiC. In the preparation of Cu_2_O–Pt/SiC, CuSO_4_·5H_2_O aqueous solution in the concentration of 0.6 mmol L^−1^ and 2 mL of methanol were introduced to the quartz cell together with Pt/SiC. The content of Cu species in the final sample was controlled by changing the irradiation time from 1 to 10 h, the resulting sample was denoted as Cu_2_O–Pt−*y*h/SiC (*y* = 1, 3, 5, 8, 10). For the synthesis of Pt/SiC/IrO_*x*_, Na_3_IrCl_6_ and NaIO_3_ aqueous solution with the respective concentration of 0.6 and 0.01 mol L^−1^ were added into the reactor to mix with Pt/SiC. The content of Ir species in the final sample was adjusted also by the irradiation time from 1 to 10 h, the resulting sample was denoted as Pt/SiC/IrO_*x*_ − *y*h (*y* = 1, 3, 5, 8, 10). After IrO_*x*_ photodeposition, the samples were washed by deionized water and ethanol for several times to remove residual iodine species. The final sample was obtained after drying in vacuum for 1 h. In order to detect the residual iodine ions, the samples were immersed in the AgNO_3_ solution. However, we did not find any precipitates AgIO_3_ or AgI in solution, indicating iodine ion can be completely washed away in the synthesis process.

### Preparation of Cu_2_O–Pt/SiC/IrO_*x*_ photocatalysts

Pt/SiC was added to the aqueous solution with CuSO_4_·5H_2_O (0.6 mmol L^−1^) and Na_3_IrCl_6_ (0.6 mmol L^−1^) followed by evacuation and irradiating with a 125 W Hg lamp. The content of Cu and Ir species in the final sample was controlled by the irradiation time from 3 to 15 h. The obtained sample was washed thoroughly with deionized water and dried in vacuum for 1 h, which was denoted as Cu_2_O–Pt-*z*h/SiC/IrO_*x*_ (*z* = 3, 5, 8, 10,12, 15).

### Preparation of Pt/WO_3_ photocatalyst

Pt/WO_3_ photocatalyst was also prepared by the photoreduction method similar to that in preparation of Pt/SiC photocatalyst with the irradiation time of 0.5 h.

### Characterization of photocatalysts

XRD patterns were recorded with Ni filtered Cu Kα radiation at 40 kV and 40 mA on a Bruker D8 Advance X-ray diffractometer. Morphology of sample was characterized by a field emission scanning electron microscopy (JSM-6700F) and TEM. TEM images were obtained at an accelerating voltage of 200 kV using a JEOL model JEM 2010 EX instrument. UV–Vis diffuse reflectance (UV–Vis DRS) spectra were obtained on a UV–Vis spectrophotometer (Cary 500) with a self-supporting sample cell, and the pure BaSO_4_ was used as a reflectance standard. Brunauer–Emmett–Teller (BET) surface area was measured with an ASAP2020M apparatus (Micromeritics Instrument Corp., USA). Nitrogen adsorption and desorption isotherms were measured at 77 K. Contents of Pt, Cu and Ir in the samples were measured using an inductively coupled plasma optical emission spectrometer (Ultima 2, HORIBA Jobin Yvon Co., France). HS-LEIS measurements were carried out on an IonTOF Qtac100 low-energy ion scattering analyser. ^4^He^+^ ions with a kinetic energy of 3 keV were applied at a low ion flux equal to 1325 pA cm^−2^, which was necessary to avoid the sputtering of surfaces. ^20^Ne^+^ ions with a kinetic energy of 5 keV were applied at a low ion flux equal to 445 pA cm^−2^. The scattering angle was 145°. XPS measurements were performed on a Quantum 2000 Scanning ESCA Microprobe (Physical Electronics) using Al Kα radiation (1846.6 eV) as the X-ray source. ^13^C NMR spectra were recorded on AVANCE III 400 MHz spectrometer using TMS as the internal standard. ^13^CO_2_ and H_2_^18^O were employed as the reactants in the isotope labelling comparison reaction instead of CO_2_ and H_2_O, respectively. After irradiation, the reaction solution was characterized with ^13^C NMR directly. FT-IR experiments were carried out on a Nicolet 670 FT-IR spectrometer at a resolution of 4 cm^−1^ and 64 scans. FT-IR experiments were performed in a home-made IR cell in conjunction with a vacuum system. The catalyst powders were first pressed into a self-supporting disk (18 mm diameter, 20 mg), and then the disk was placed in the sample cell, which allowed the disk to move vertically along the cell tube. Prior to the FT-IR measurements, the disk was treated under a dynamic vacuum (10^−4^ Torr) at 473 K for 2 h. After cooling the disk to room temperature, CO_2_ was introduced into the cell via the septum with a syringe. Photoluminescence excitation spectra was recorded on a FL/FS920 spectrofluorimeter (Edinburgh Instruments) fluorescence spectrometer at room temperature; the excitation wavelength is 375 nm.

### CO_2_ photoreduction apparatus and reactions

The spatially separated Z-scheme system includes a CO_2_-reduction chamber and an O_2_-generation chamber that are divided by a Nafion membrane (The circular Nafion membrane was Fe-ion exchanged before used in the reactor.) (Supplementary Fig. 6). The cubage of each reaction chamber is 300 mL. The aqueous solution containing 50 mg photocatalyst (SiC, Pt/SiC, Cu_2_O–Pt/SiC, Pt/SiC/IrO_*x*_ or Cu_2_O–Pt/SiC/IrO_*x*_) and 2 mM FeCl_2_ and another aqueous solution containing 100 mg Pt/WO_3_ and 2 mM FeCl_3_ were added to the two reaction chambers, respectively. The pH of solutions was adjusted to 2.3 by adding hydrochloric acid to prevent hydrolysis of the iron ions. Prior to irradiation, ultra-pure Ar (99.9995 v%) gas was bubbled through the solution to purge any dissolved air in the O_2_-generation compartment and filled to atmospheric pressure, while ultra-pure CO_2_ gas was bubbled through the solution in the CO_2_-reduction chamber and filled to atmospheric pressure. During the photoreaction, the solution in each chamber was stirred and irradiated with a 300 W xenon (Xe) lamp. The lamp with an optical filter (*λ* ≥ 420 nm) was vertically placed at equal distance from each chamber, which makes both solutions to receive the same amount of visible light intensity. The solution in the CO_2_-reduction chamber was sampled every 2 h and analysed by ion chromatography (IC, Thermofisher ICS 1100) after filtering catalyst. The gaseous products in O_2_ evolution chamber were sampled every 2 h by an off-line sampling syringe (0.5 mL) and then analysed by the gas chromatography (GC, Agilent 7890B, TCD detector) using ultra-pure Ar as the carrier gas.

### Photoelectrochemical measurements

Photoelectrochemical measurements were carried out with a BAS Epsilon workstation using a standard three-electrode electrochemical cell with a working electrode, a platinum foil as the counter electrode, and a saturated Ag/AgCl electrode as the reference. A sodium sulfate solution (0.2 M) was used as the electrolyte, and a 300 W Xe lamp (*λ* = 320–780 nm) as the light source. The working electrode was prepared by FTO glass pieces, which was cleaned by sonication in cleanout fluid, acetone and ethanol in sequence prior to use. The photocatalyst was dispersed in ethanol under sonication to form a suspension. A photocatalyst film was fabricated by spreading the suspension onto the conductive surface of the FTO glass.

## Supplementary information

Supplementary Information

Peer Review File

## Data Availability

The data that support the findings of this study are available from the corresponding author upon reasonable request. Source data are provided with this paper.

## Source data

Source Data
