# Supplementary material for: Direct and indirect Z-scheme heterostructure-coupled photosystem enabling cooperation of CO2 reduction and H2O oxidation
Source: Nat Commun. 2020 Jun 16;11:3043. doi: 10.1038/s41467-020-16742-3 (PMC7297725; doi:10.1038/s41467-020-16742-3)
Supplement: Supplementary file 1 — Supplementary Information [file 41467_2020_16742_MOESM1_ESM.pdf]

Supplementary Information

Direct and indirect Z-scheme heterostructure-coupled photosystem enabling cooperation of CO_2 reduction and H_2O oxidation

Ying Wang,[1,2] Xiaotong Shang,[1] Jinni Shen,[1] Zizhong Zhang,*[,1] Debao Wang,[2] Jinjin Lin,[1] Jeffrey C. S. Wu,*[,3] Xianzhi Fu,[1] Xuxu Wang,*[,1] and Can Li*[,4]

1. State Key Laboratory of Photocatalysis on Energy and Environment, Research Institute of Photocatalysis, College of Chemistry, Fuzhou University, P. R. China.
2. Key Laboratory of Optic-electric Sensing and Analytical Chemistry for Life Science, College of Chemistry and Molecular Engineering, Qingdao University of Science and Technology, Qingdao 266042, P. R. China.
3. Department of Chemical Engineering, National Taiwan University, Taipei 10617, China.
4. State Key Laboratory of Catalysis, Dalian Institute of Chemical Physics, Chinese Academy of Sciences, Dalian 116023, China.

Supplementary Figures

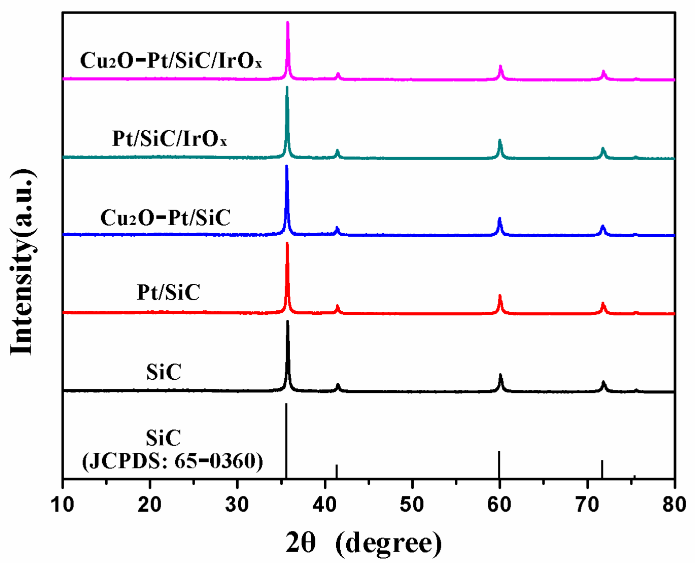

Supplementary Fig. 1 XRD patterns of SiC, Pt/SiC, Cu_2O-Pt/SiC, Pt/SiC/IrO_x and Cu_2O-Pt/SiC/IrO_x catalysts.

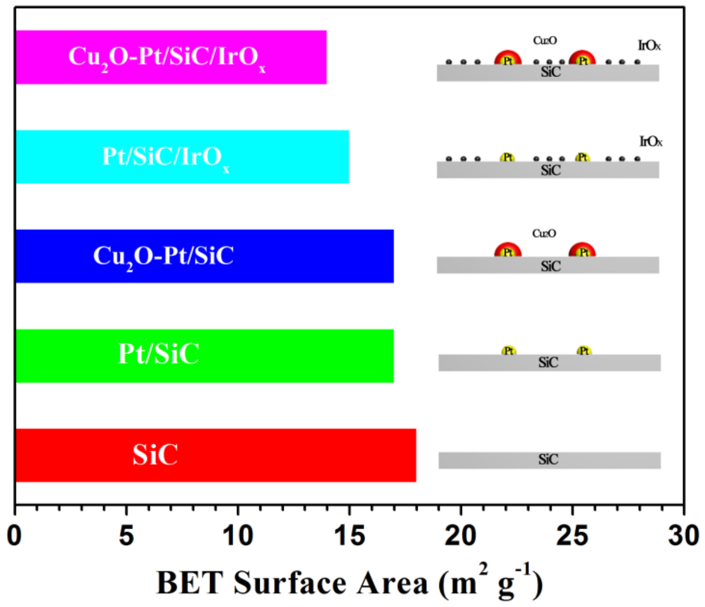

Supplementary Fig. 2 BET surface area of SiC, Pt/SiC, Cu_2O-Pt/SiC, Pt/SiC/IrO_x and Cu_2O-Pt/SiC/IrO_x catalysts.

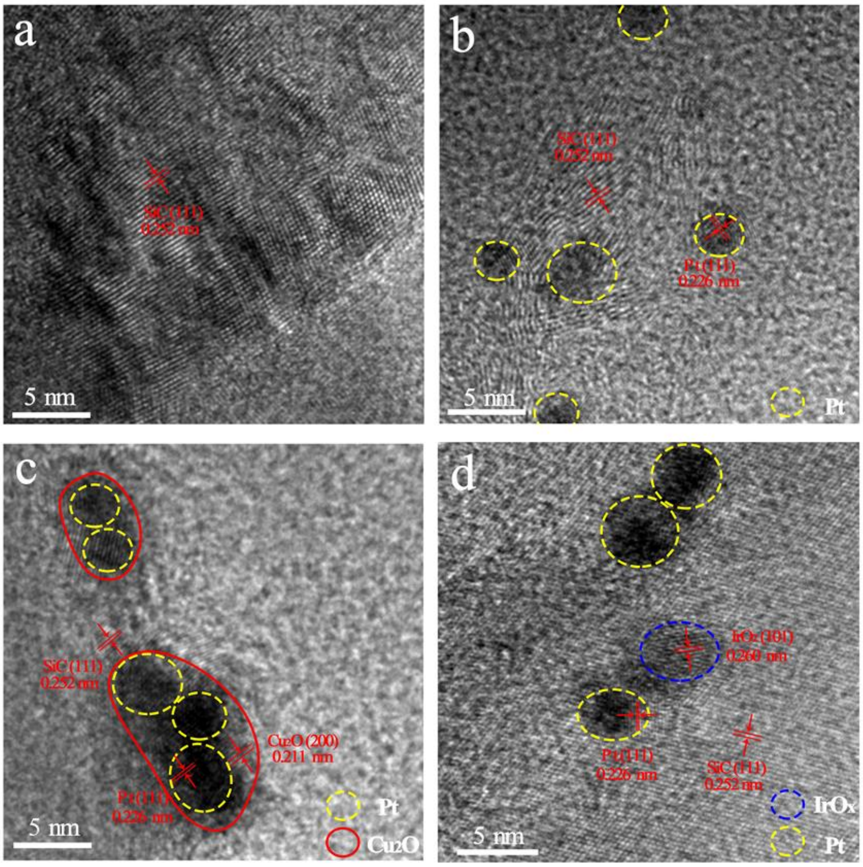

Supplementary Fig. 3 Cocatalysts distribution on photocatalysts. TEM images of a) SiC, b) Pt/SiC, c) Cu_2O-Pt/SiC and d) Pt/SiC/IrO_x.

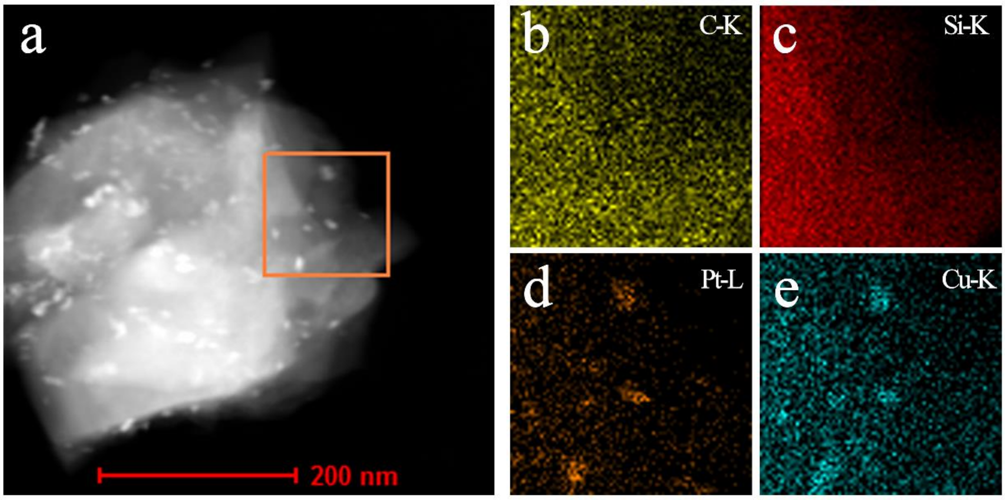

Supplementary Fig. 4 Consistent distribution of Cu_2O and Pt on Cu_2O-Pt/SiC. a) STEM image and corresponding EDS mapping profiles of Cu_2O-Pt/SiC for b) C-K, c) Si-K, d) Pt-L, and e) Cu-K.

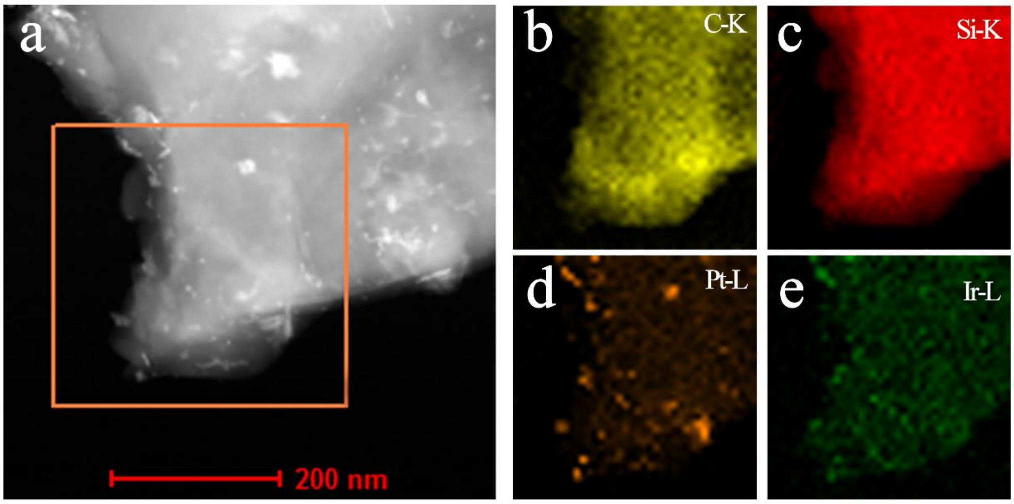

Supplementary Fig. 5 Separated distribution of Pt and IrOx on Pt/SiC/IrO$_x$. a) STEM image and corresponding EDS mapping profiles of Pt/SiC/IrO$_x$ for b) C-K, c) Si-K, d) Pt-L, and e) Ir-L.

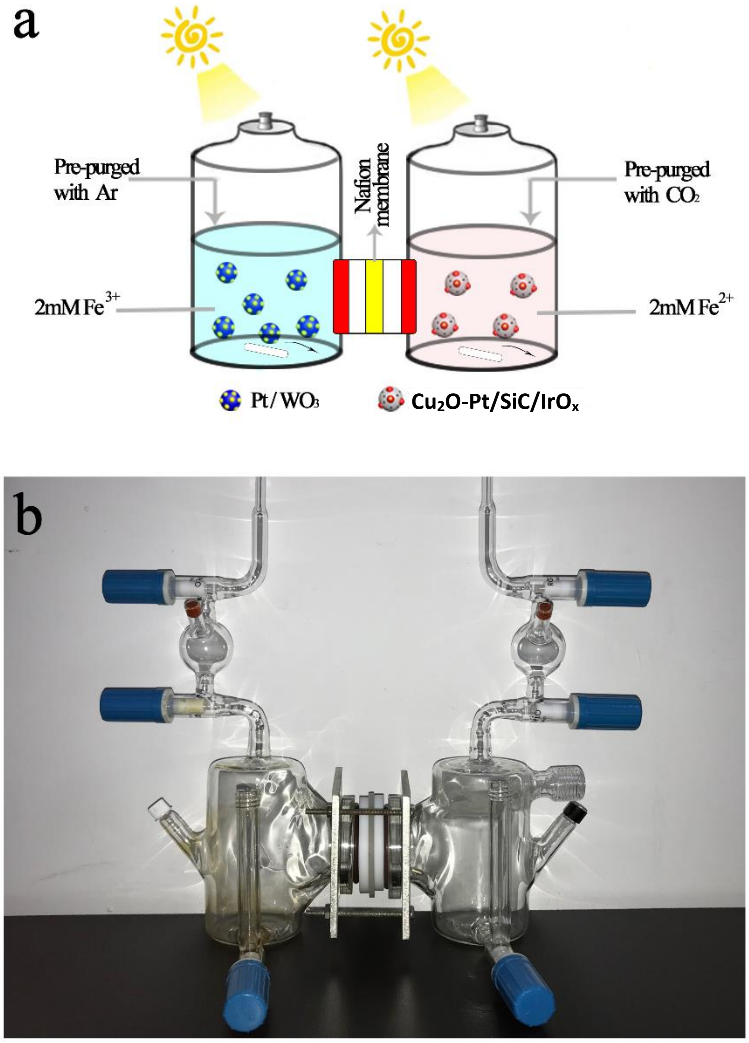

Supplementary Fig. 6 Diagrams of spatially-separated Z-scheme. a) Schematic diagram of the spatially separated Z-scheme system. b) Diagram of experimental spatially-separated Z-scheme device.

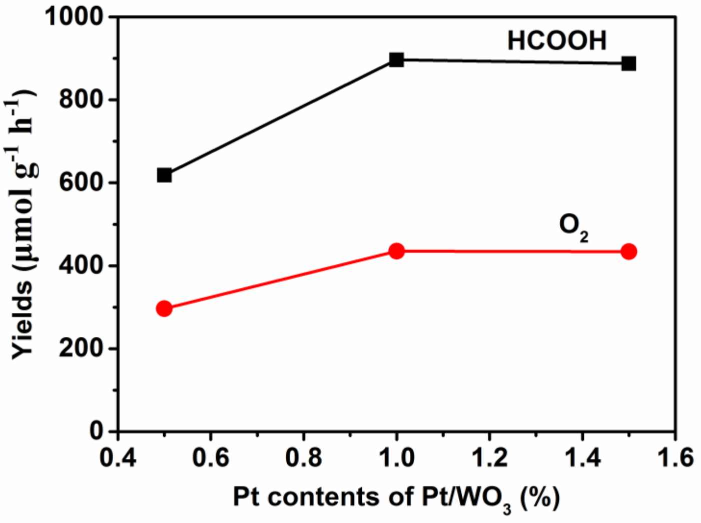

Supplementary Fig. 7 The effect of Pt contents of Pt/WO_3 on the O_2 and HCOOH evolution in the separated reaction system.

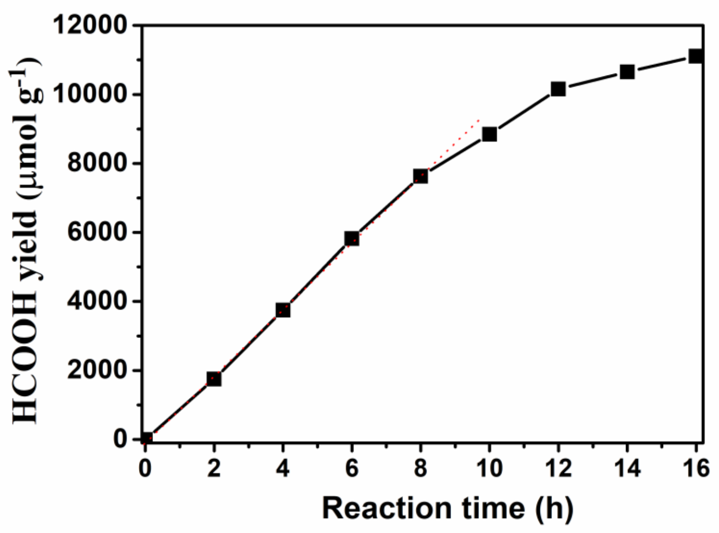

Supplementary Fig. 8 The HCOOH evolution with the reaction time prolonging to 16 h.

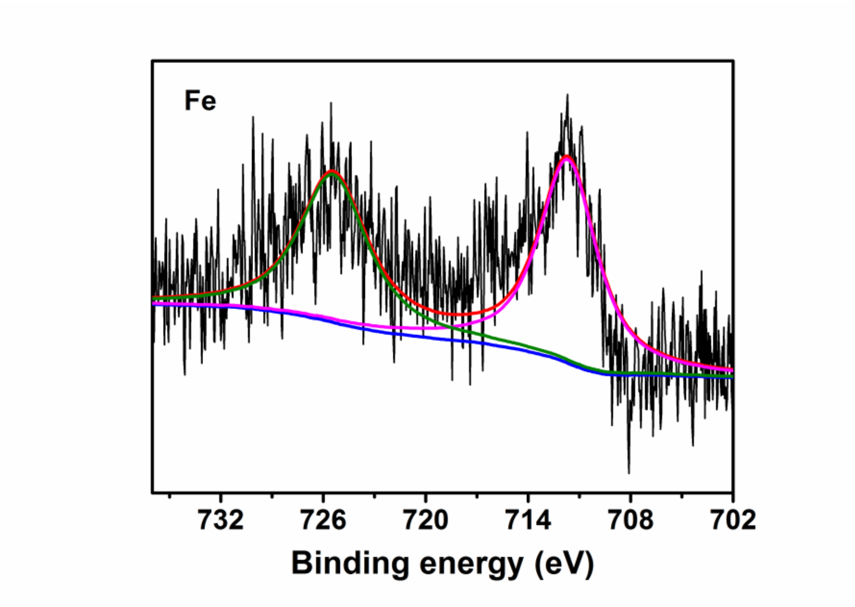

Supplementary Fig. 9 The Fe 2p XPS spectrum of the Cu_2O-Pt/SiC/IrO_x sample after 16 h reaction.

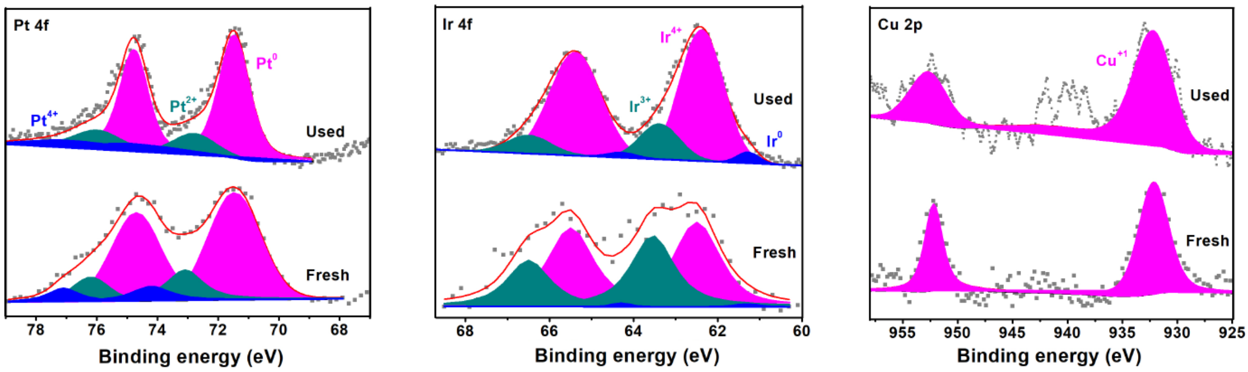

Supplementary Fig. 10 The XPS spectra of the Cu_2O-Pt/SiC/IrO_x sample before and after cycle reaction.

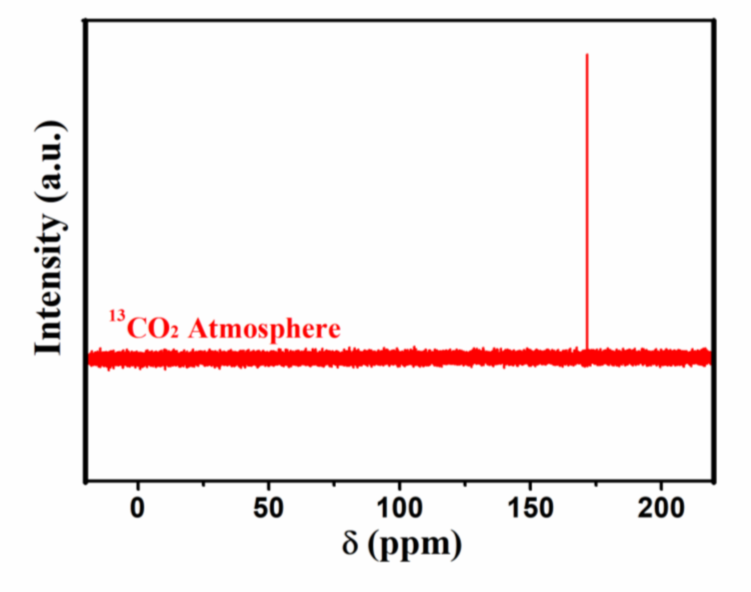

Supplementary Fig. 11 ^{13}C NMR spectra of the photocatalytic products at $^{13}CO_2$ atmosphere over Cu_2O-Pt/SiC/IrO_x.

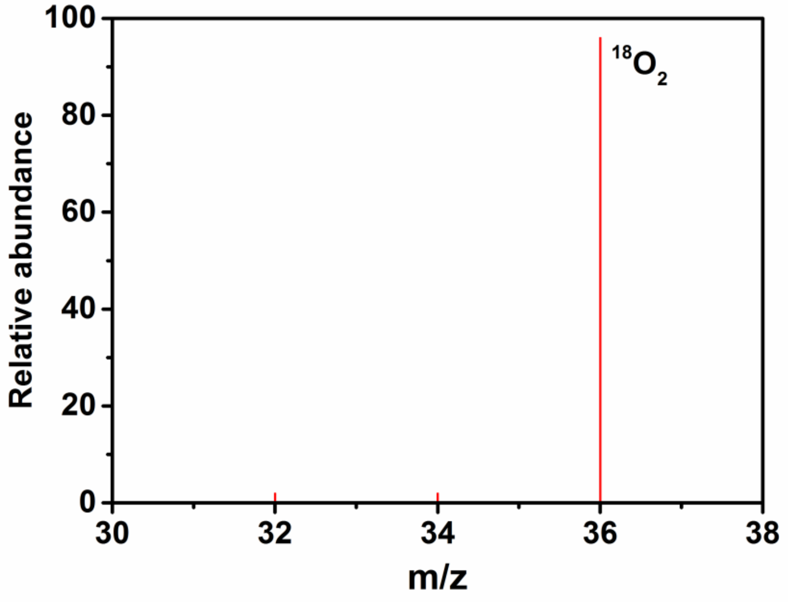

Supplementary Fig. 12 The mass spectra of product O_2 over Pt/WO_3 in the 2 ml $H_2{}^{18}O$ solution.

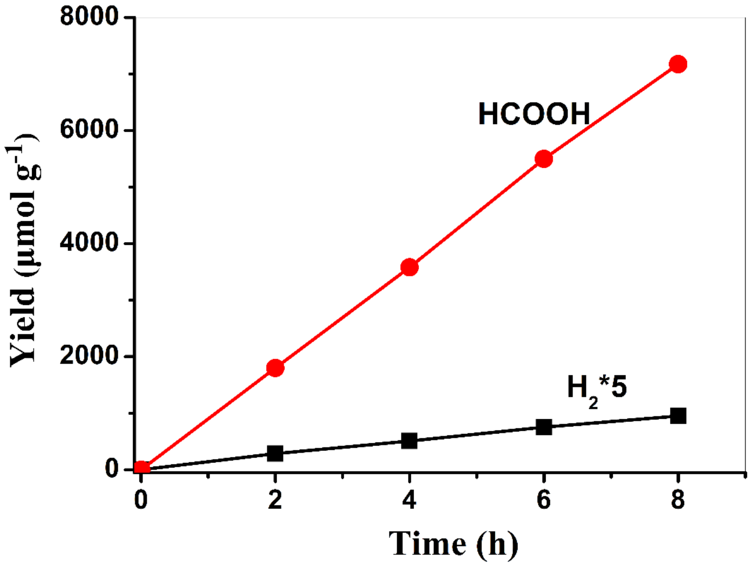

Supplementary Fig. 13 HCOOH and H_2 evolution over Cu_2O-Pt/SiC/IrO_x in spatially-separated Z-scheme reactor under visible light.

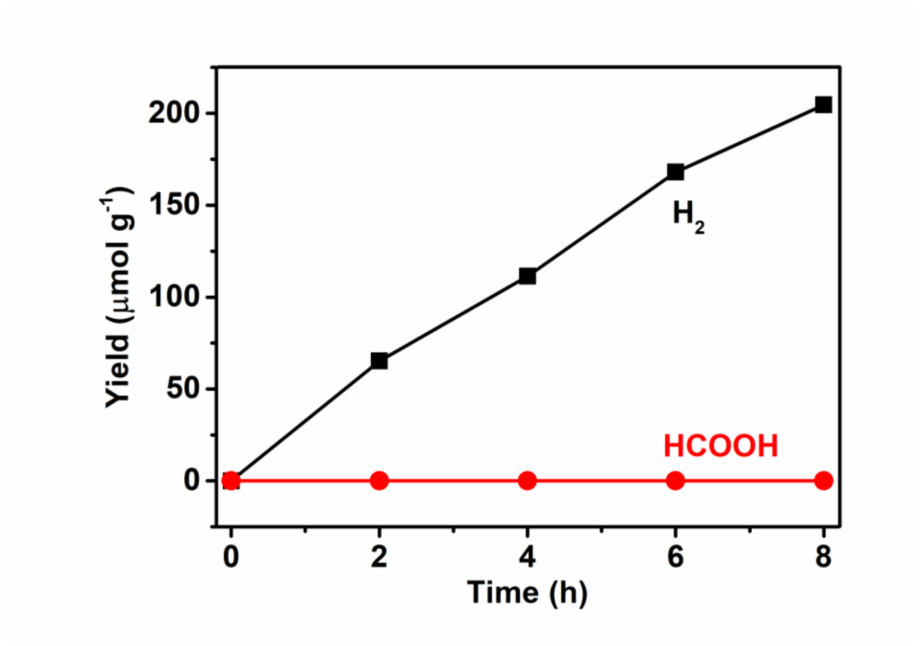

Supplementary Fig. 14 HCOOH and H_2 evolution over Cu_2O-Pt/SiC/IrO_x performed a blank experiment without any CO_2 under visible light.

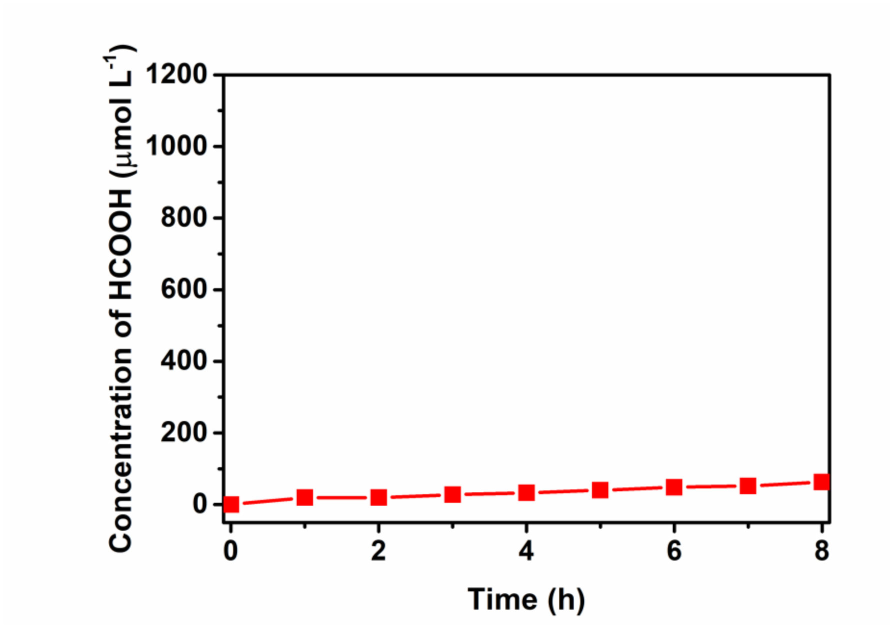

Supplementary Fig. 15 Permeation of HCOOH from the HCOOH solution (1200 μmol L^{-1}) to pure H_2O across the Nafion membrane.

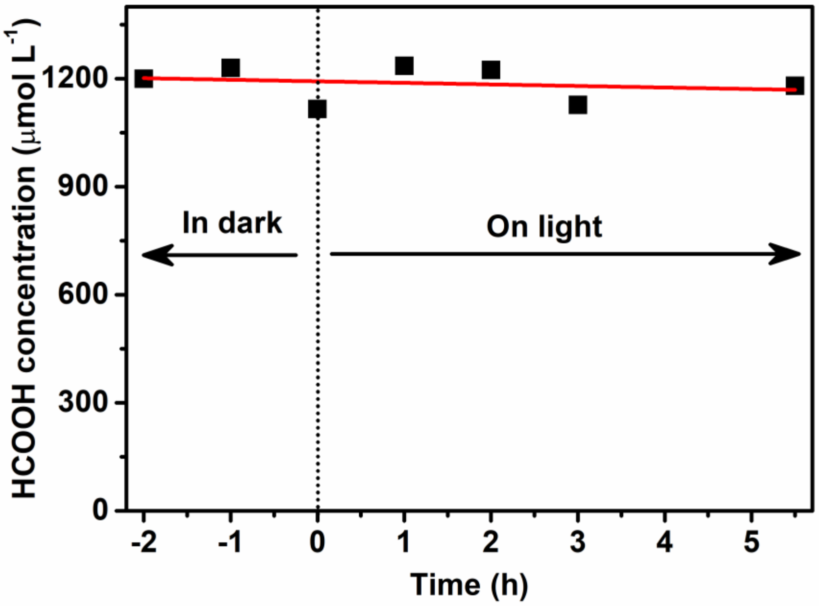

Supplementary Fig. 16 The oxidation of HCOOH solution (1200 μmol L^{-1}) containing 2 mmol L^{-1} $FeCl_2$/$FeCl_3$ in darkness and under visible light irradiation.

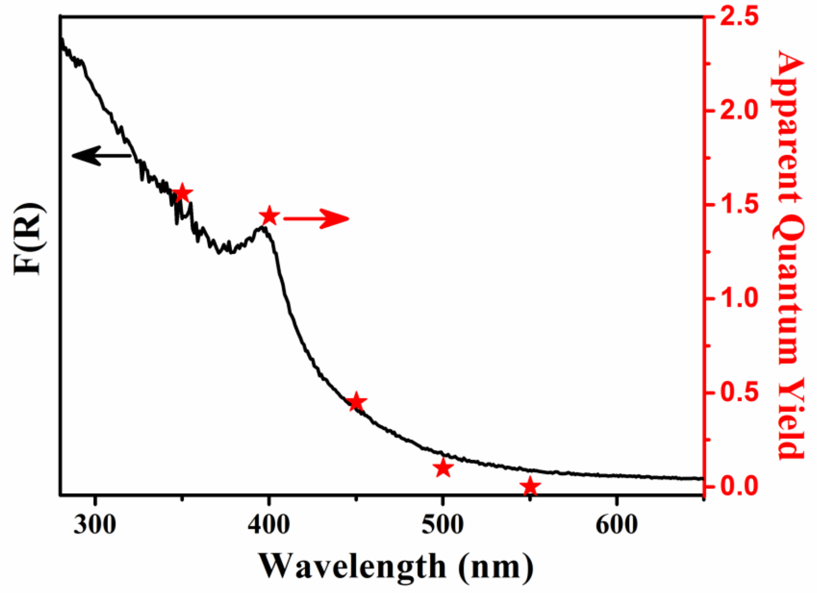

Supplementary Fig. 17 The apparent quantum yield of HCOOH evolution with the wavelength of irradiation light for the separated reaction system.

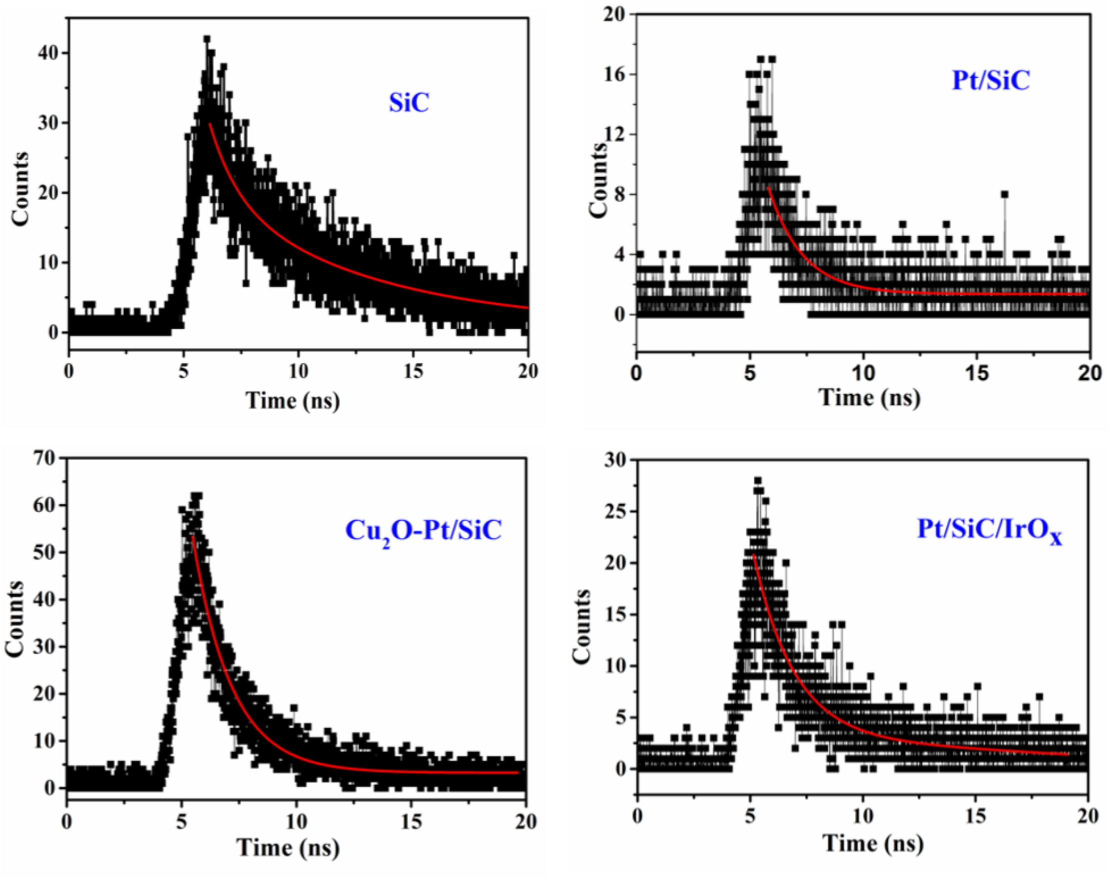

Supplementary Fig. 18 Time-resolved photoluminescence spectroscopy for the SiC, Pt/SiC, Cu_2O-Pt/SiC and Pt/SiC/IrO_x catalysts

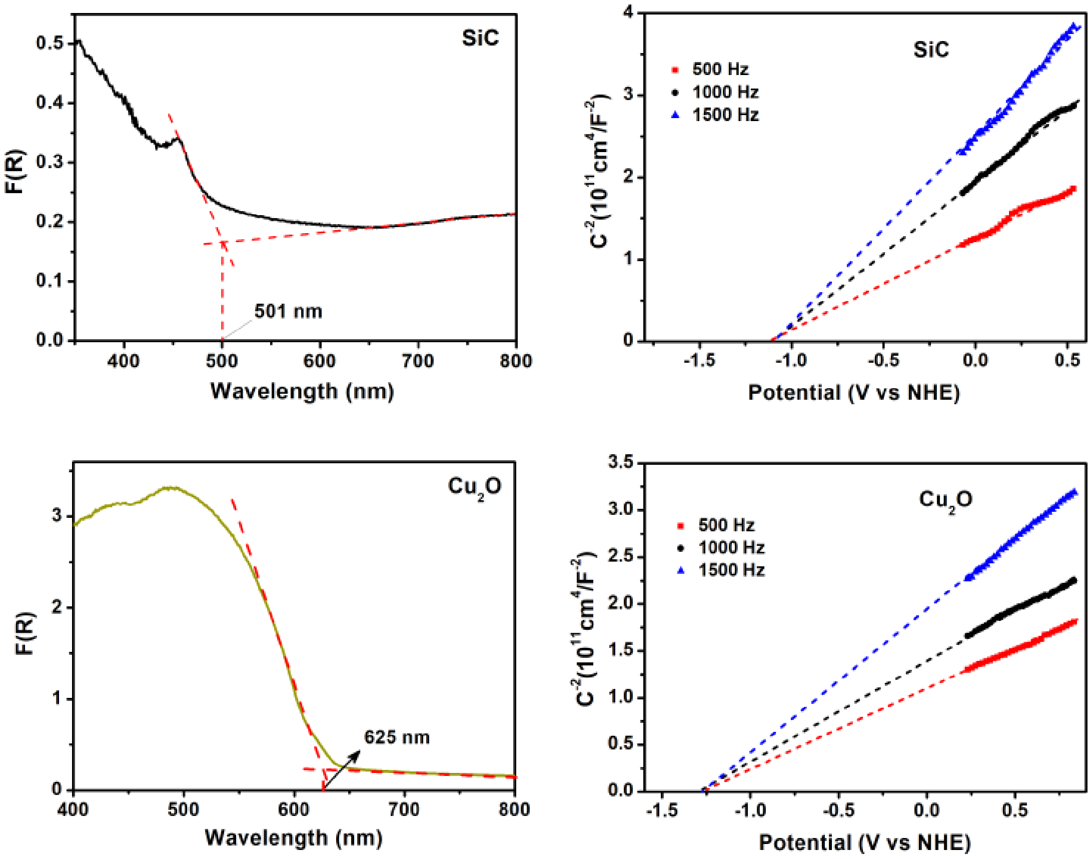

Supplementary Fig. 19 Band Structure of SiC and Cu_2O. DRS spectra and Mott-Schottky plots of SiC and Cu_2O catalysts.

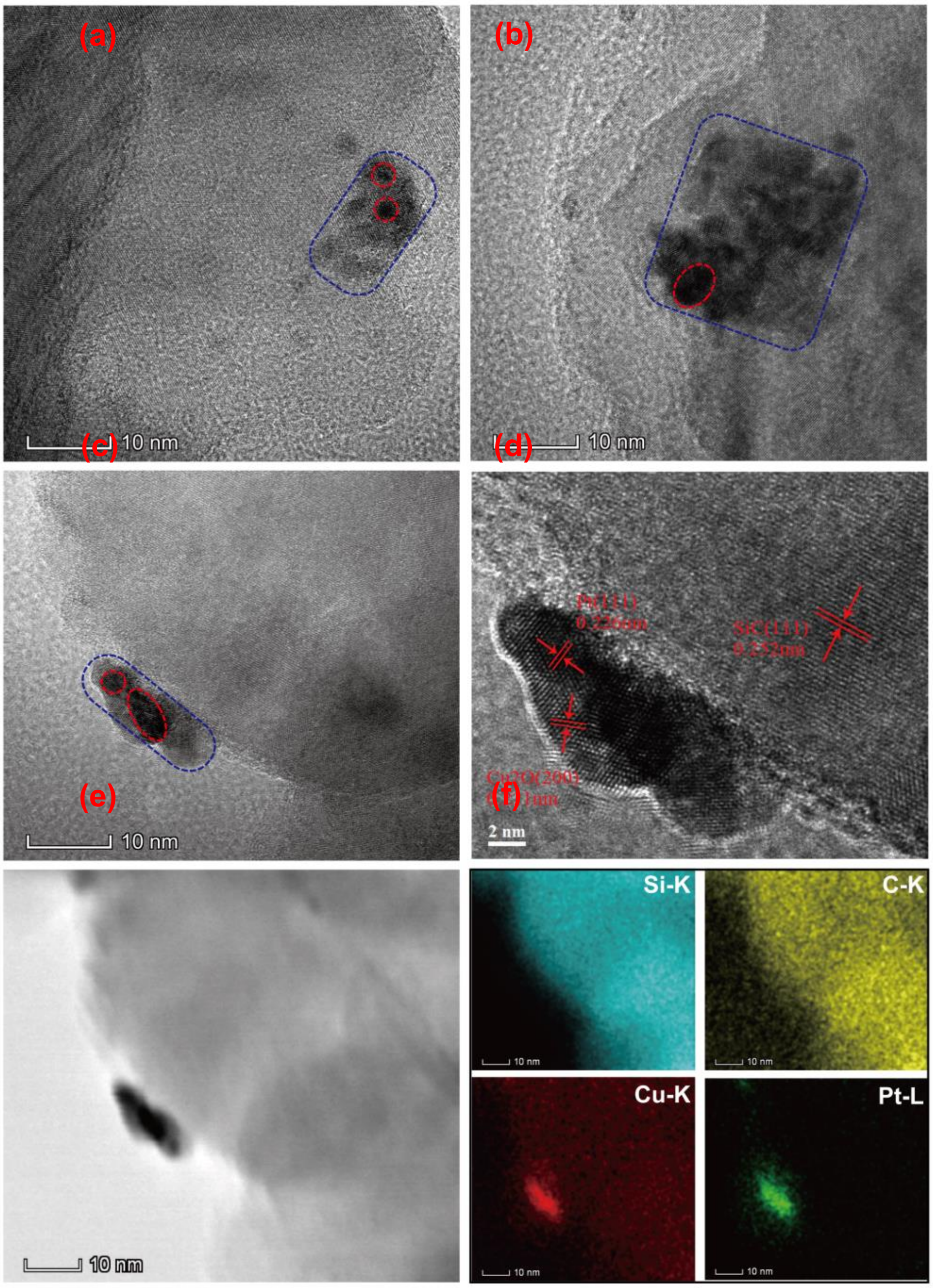

Supplementary Fig. 20 Intimate contact of cocatalysts Pt and Cu_2O. TEM images (a, b, c), HRTEM images (d), STEM images (e), EDX elemental mapping images (f) of Cu_2O/SiC photodeposited with Pt, the Pt nanoparticles were marked with red dotted circle and Cu_2O were marked with blue dotted circle.

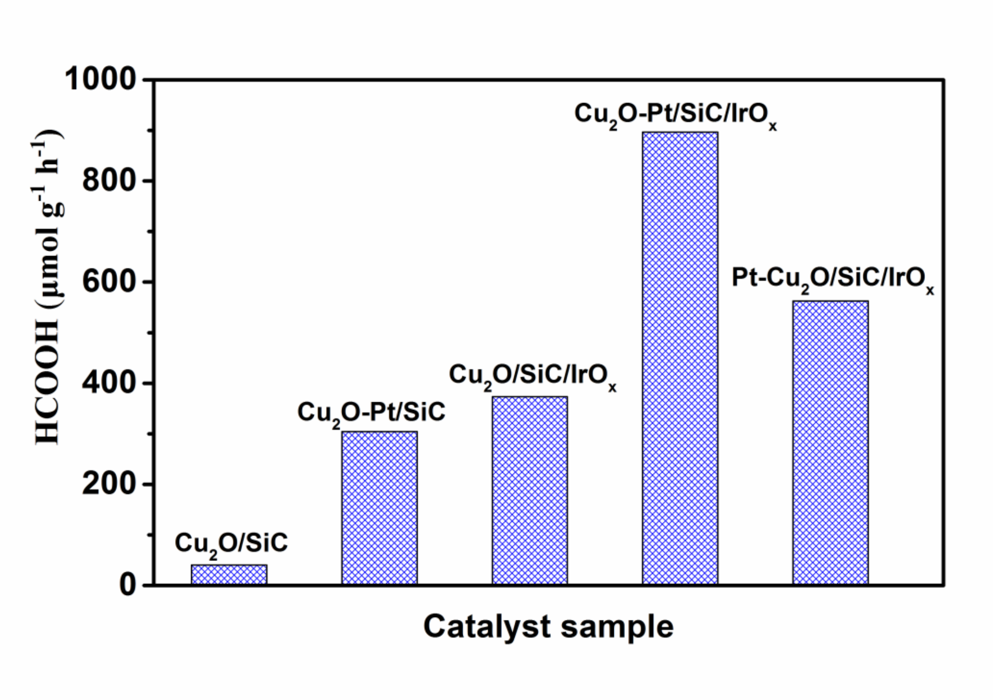

Supplementary Fig. 21 The comparison of HCOOH evolution over the various photocatalysts.

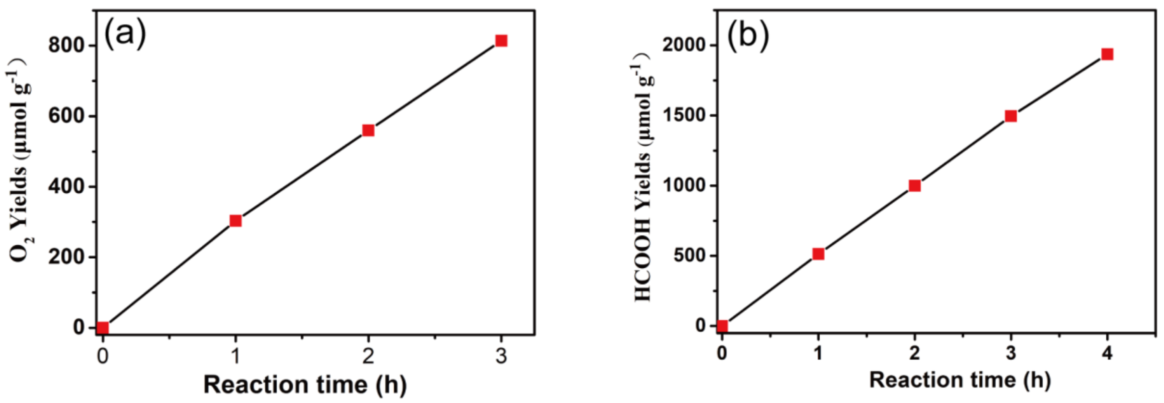

Supplementary Fig. 22 Controlled experiments in one-pot reactor. (a) O_2 evolution with reaction time in the solution with Pt/WO_3, Fe^{3+} and H_2O. (b) HCOOH production with reaction time in the solution with Cu_2O-Pt/SiC/IrO_x, Fe^{2+}, H_2O and CO_2, with one-pot reactor under visible light irradiation.

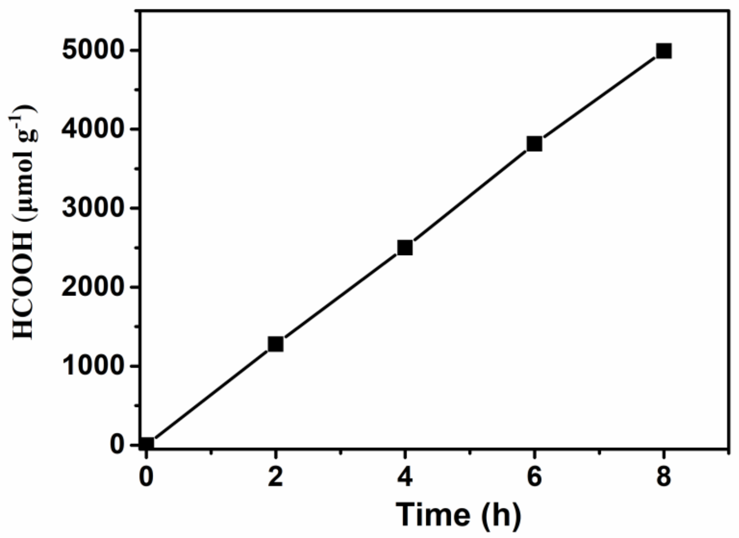

Supplementary Fig. 23 The HCOOH evolution in the separated reaction system with Fe^{2+}/Fe^{3+} even dispersion in both cells.

Supplementary Tables

Supplementary Table 1. Contents of Pt, Cu and Ir in SiC, Pt-xh/SiC, Cu_2O-Pt-yh/SiC, Pt/SiC/IrO_x-yh and Cu_2O-Pt-zh/SiC/IrO_x series of catalysts[a] and the their photocatalytic HCOOH evolution under visible light (≥420 nm).

Photocatalyst	Pt content (wt%)	Cu content (wt%)	Ir content (wt%)	HCOOH (μmol g^{-1} h^{-1})
SiC	—	—	—	24.1
Pt-0.5h/SiC	0.83	—	—	41.1
Pt-1.0h/SiC	1.3	—	—	57.8
Pt-1.5h/SiC	1.9	—	—	43.6
Pt-2.0h/SiC	2.6	—	—	31.7
Cu_2O-Pt-1h/SiC	1.3	0.52	—	163.2
Cu_2O-Pt-3h/SiC	1.3	1.2	—	211.2
Cu_2O-Pt-5h/SiC	1.3	1.9	—	304.6
Cu_2O-Pt-8h/SiC	1.3	2.5	—	238.7
Cu_2O-Pt-10h/SiC	1.3	2.7	—	173.6
Pt/SiC/IrO_x-1h	1.3	—	0.87	140.4
Pt/SiC/IrO_x-3h	1.3	—	1.6	251.3
Pt/SiC/IrO_x-5h	1.3	—	2.3	472.0
Pt/SiC/IrO_x-8h	1.3	—	2.6	462.9
Pt/SiC/IrO_x-10h	1.3	—	2.8	250.1
Cu_2O-Pt-3h/SiC/IrO_x	1.3	0.95	1.1	369.2
Cu_2O-Pt-5h/SiC/IrO_x	1.3	1.3	1.6	529.2
Cu_2O-Pt-8h/SiC/IrO_x	1.3	1.8	2.2	896.7
Cu_2O-Pt-10h/SiC/IrO_x	1.3	2.1	2.6	654.7
Cu_2O-Pt-12h/SiC/IrO_x	1.3	2.3	3.1	518.9
Cu_2O-Pt-15h/SiC/IrO_x	1.3	2.7	3.2	422.0

[a] Measured by ICP-MS.

Supplementary Table 2. Pt chemical state ratio (%) calculated from the Pt 4f XPS spectra of various photocatalyst samples.

Sample	Pt^0	Pt^{2+}	Pt^{4+}	Sum(Pt^{2+}+Pt^{4+})
SiC	—	—	—	—
Pt/SiC	67.5%	24.6%	7.9%	32.5%
Cu_2O-Pt/SiC	66.8%	19.6%	13.6%	33.2%
Pt/SiC/IrOx	72.5%	20.0%	7.5%	27.5%
Cu_2O-Pt/SiC/IrO_x	74.1%	16.5%	9.4%	25.9%

Supplementary Table 3. Visible light photocatalytic CO_2 reduction to HCOOH over various semiconductor photocatalysts under similar conditions.

Photocatalyst	Light source	HCOOH (umo*g^{-1}*h^{-1})	Reference
Binuclear Ru(II) complex/C_3N_4	λ ⩾ 400 nm	133	1
[Ru-dcbpy]/N-Ta_2O_5	410 ⩽ λ ⩽ 750 nm	70	2
Cu/$BiYO_3$	Visible light	170	3
Ru complex/C_3N_4	λ ⩾ 400 nm	46	4
Ru complex /Ag/$CaTaO_2N$	Visible light	5	5
C and Fe co-doped $LaCoO_3$	λ ⩾ 400 nm	128	6
((Mo-Bi)Sx/Meso CdS	420 ⩽ λ ⩽ 750 nm	208	7
TiO_2	λ ⩾ 340 nm	1.2	8
CdS	λ ⩾ 320 nm	81.5	9

Supplementary Table 4. The contents of Ir^{3+}, Ir^{4+} and Ir^{0} in Cu_2O-Pt/SiC/IrO_x sample before and after cycle reaction.

	Ir^{3+}	Ir^{4+}	Ir^{0}
Content in used sample (%)	16.2	81.0	2.8
Content in fresh sample (%)	40.4	58.8	0.8

Supplementary Table 5. HCOOH evolution in the one-pot reaction system

Samples	One-pot reactor (no Fe^{2+}& Fe^{3+}) (μmol g^{-1} h^{-1})	One-pot reactor (with Fe^{2+}& Fe^{3+}) (μmol g^{-1} h^{-1})	Separated reactor (with Fe^{2+}& Fe^{3+}) (μmol g^{-1} h^{-1})
SiC	0.63	1.70	24.06
Pt/SiC	1.37	3.55	57.74
Cu_2O-Pt/SiC	6.83	18.65	304.65
Pt/SiC/IrO_x	11.09	30.39	472
Cu_2O-Pt/SiC/IrO_x	22.71	61.54	896.71

Supplementary Notes

Supplementary Note 1

Photodeposition of co-catalyst species on catalysts. Supplementary Table 1 shows the contents of Pt, Cu and Ir in SiC, Pt/SiC, Cu_2O-Pt/SiC, Pt/SiC/IrO_x and Cu_2O-Pt/SiC/IrO_x series of catalysts prepared by changing the irradiation time for photodeposition of co-catalyst species. The increase in the irradiation time for the deposition of Pt species from 0.5 to 2.0 h increases the content of Pt from 0.83 to 2.6 wt%. The contents of Pt in Cu_2O-Pt/SiC, Pt/SiC/IrO_x and Cu_2O-Pt/SiC/IrO_x series of catalysts are ~1.3 wt%. Consistent with that of Pt, the contents Cu_2O and IrO_x are also enhanced along with the irradiation time.

Supplementary Table 1 also shows the photocatalytic HCOOH evolution rate of Pt/SiC, Cu_2O-Pt/SiC, Pt/SiC/IrO_x and Cu_2O-Pt/SiC/IrO_x series of catalysts. Pt-1.0 h/SiC with the highest activity acts as the substrate for further photodeposition to synthesize Cu_2O-Pt/SiC, Pt/SiC/IrO_x and Cu_2O-Pt/SiC/IrO_x. According to the HCOOH evolution rate, the optimal loading amount of Cu_2O and IrO_x are 1.9 wt% and 2.3 wt%, respectively. In keeping with independent cases, the optimal loading amount of Cu_2O and IrO_x in Cu_2O-Pt/SiC/IrO_x approach 1.8 wt% and 2.2 wt%. From the above, the optimal deposition amount is ascertained to be 1.3, 1.8 and 2.3 wt% for Pt, Cu_2O and IrO_x, respectively.

Supplementary Note 2

Crystallographic structure of photocatalysts. The crystallographic structure of the as-prepared samples was studied by XRD, as shown in Supplementary Fig. 1. Five peaks ascribed to cubic phase 3C-SiC (JCPDS No. 65-0360) were observed for all samples. Namely, the peaks at 35.6°, 41.4°, 60.0°, 71.8° and 75.5° correspond to the (1 1 1), (2 0 0), (2 2 0), (3 1 1) and (2 2 2) crystal planes, respectively, of 3C-SiC. Among all the samples, no diffraction peaks for Pt, Cu_2O or IrO_x species were observed, which may result from the low loaded content or fine dispersion of metal species on the surface of SiC.

Supplementary Note 3

BET surface area of photocatalysts. Supplementary Fig. 2 shows the BET surface area of SiC, Pt/SiC, Cu_2O-Pt/SiC, Pt/SiC/IrO_x and Cu_2O-Pt/SiC/IrO_x catalysts. The slight variation among these samples can be explained by the co-catalyst distribution as shown at right side in Supplementary Fig. 2. Bare SiC has the largest BET surface area because that loading of co-catalysts may block the pore structure. The BET surface area of Cu_2O-Pt/SiC changes little comparing with the Pt/SiC. However, the separated deposition site of IrO_x and Cu_2O-Pt can further decrease the BET surface area in Pt/SiC/IrO_x as well as Cu_2O-Pt/SiC/IrO_x.

Supplementary Note 4

HRTEM images and EDS mapping profiles of photocatalysts. Supplementary Fig. 3 shows the HRTEM images of SiC, Pt/SiC, Cu_2O-Pt/SiC and Pt/SiC/IrO_x. Only one lattice fringe of 0.252 nm is found in SiC (Supplementary Fig. 3a), which corresponds to the (111) facet of SiC. After the photodeposition of Pt, Pt nanoparticles ranging from 2 to 4 nm are observed on account of their high contrasity in HRTEM image (Supplementary Fig. 3b) and circled with yellow dash line. Besides that of SiC, another measured lattice fringe of 0.226 nm in yellow dotted circle can be attributed to the (111) facet of Pt. Intimate contact structure Cu_2O-Pt is obviously displayed when Cu_2O has been further deposited on Pt/SiC, and the Cu_2O is marked with the red circle in Supplementary Fig. 3c. The additional lattice fringe of 0.211 nm is assigned to the (200) facet of Cu_2O. In addition, the EDS mapping profiles of Cu_2O-Pt/SiC shown in Supplementary Fig. 4 also demonstrates the Cu_2O-Pt structure, according to the distribution of Pt overlaps with that of Cu. When IrO_x is introduced on the surface of Pt/SiC instead of Cu_2O, the isolated Pt (marked with yellow dotted circle) and IrO_x (marked with blue dotted circle) nanoparticles are visible in HRTEM image (Supplementary Fig. 3d). The lattice fringe of 0.260 nm is assigned to the (101) facet of IrO_2. In line with the observation in HRTEM image, the EDS mapping profiles of Pt/SiC/IrO_x (Supplementary Fig. 5) illustrates that Pt and IrO_x nanoparticles are distributed at different positions on the surface of SiC.

Supplementary References

1. Kuriki, R. et al. (2016). Nature-inspired, highly durable CO_2 reduction system consisting of a binuclear ruthenium (II) complex and an organic semiconductor using visible light. *J. Am. Chem. Soc.* **138**, 5159-5170 (2016).
2. Sato, S., Morikawa, T., Saeki, S., Kajino, T. & Motohiro, T. Visible-light-induced selective CO_2 reduction utilizing a ruthenium complex Electrocatalyst linked to ap-type nitrogen-doped Ta_2O_5 semiconductor. *Angew. Chem. Int. Ed*. **49**, 5101-5105 (2010).
3. Su, T., Tian, H., Qin, Z. & Ji, H. Preparation and characterization of Cu modified $BiYO_3$ for carbon dioxide reduction to formic acid. *Appl. Catal. B: Environ.* **202**, 364-373 (2017).
4. Maeda, K., Kuriki, R., Zhang, M., Wang, X. & Ishitani, O. The effect of the pore-wall structure of carbon nitride on photocatalytic CO_2 reduction under visible light. *J. Mater. Chem. A* **2**, 15146-15151 (2014).
5. Yoshitomi, F., Sekizawa, K., Maeda, K. & Ishitani, O. Selective formic acid production via CO_2 reduction with visible light using a hybrid of a perovskite tantalum oxynitride and a binuclear ruthenium (II) complex. *ACS Appl. Mater. Interfaces*, **7**, 13092-13097 (2015).
6. Jia, L., Li, J. & Fang, W. Enhanced visible-light active C and Fe co-doped $LaCoO_3$ for reduction of carbon dioxide. *Catal. Commun.* ***2009**, 11*, 87-90 (2009).
7. Zhou, B. et al. Mo–Bi–Cd Ternary Metal Chalcogenides: Highly Efficient Photocatalyst for CO_2 Reduction to Formic Acid Under Visible Light. *ACS Sustain. Chem. Eng*. **6**, 5754-5759 (2018).
8. Kaneco, S., Kurimoto, H., Ohta, K., Mizuno, T. & Saji, A. Photocatalytic reduction of CO_2 using TiO_2 powders in liquid CO_2 medium. *Photochem. Photobiol. A* **109**, 59-63 (1997).
9. Kisch, H. & Lutz, P. Photoreduction of bicarbonate catalyzed by supported cadmium sulfide. *Photochem. Photobiol. Sci.* **1**, 240-245 (2002).
